# Kidins220 and Aiolos promote thymic iNKT cell development by reducing TCR signals

**DOI:** 10.1126/sciadv.adj2802

**Published:** 2024-03-15

**Authors:** Laurenz A. Herr, Gina J. Fiala, Sagar, Anna-Maria Schaffer, Jonas F. Hummel, Marina Zintchenko, Katrin Raute, Rubí M.-H. Velasco Cárdenas, Beate Heizmann, Karolina Ebert, Kerstin Fehrenbach, Iga Janowska, Susan Chan, Yakup Tanriver, Susana Minguet, Wolfgang W. Schamel

**Affiliations:** ^1^ Signaling Research Centers BIOSS and CIBSS; University of Freiburg, Freiburg, Germany.; ^2^ Department of Immunology, Faculty of Biology, University of Freiburg, Freiburg, Germany.; ^3^ Centre for Chronic Immunodeficiency (CCI), Medical Center, University of Freiburg, Freiburg, Germany.; ^4^ Spemann Graduate School of Biology and Medicine (SGBM), University of Freiburg, Freiburg, Germany.; ^5^ Department of Medicine II (Gastroenterology, Hepatology, Endocrinology, and Infectious Diseases), Freiburg University Medical Center, Faculty of Medicine, University of Freiburg, Freiburg, Germany.; ^6^ Institute of Medical Microbiology and Hygiene, Medical Center, University of Freiburg, Germany.; ^7^ Institut de Génétique et de Biologie Moléculaire et Cellulaire (IGBMC), INSERM U1258, CNRS UMR7104, Université de Strasbourg, Illkirch, France.; ^8^ Department of Medicine IV: Nephrology and Primary Care, Medical Center, University of Freiburg, Freiburg, Germany.

## Abstract

Development of T cells is controlled by the signal strength of the TCR. The scaffold protein kinase D–interacting substrate of 220 kilodalton (Kidins220) binds to the TCR; however, its role in T cell development was unknown. Here, we show that T cell–specific Kidins220 knockout (T-KO) mice have strongly reduced invariant natural killer T (iNKT) cell numbers and modest decreases in conventional T cells. Enhanced apoptosis due to increased TCR signaling in T-KO iNKT thymocytes of developmental stages 2 and 3 shows that Kidins220 down-regulates TCR signaling at these stages. scRNA-seq  indicated that the transcription factor Aiolos is down-regulated in Kidins220-deficient iNKT cells. Analysis of an Aiolos KO demonstrated that Aiolos is a downstream effector of Kidins220 during iNKT cell development. In the periphery, T-KO iNKT cells show reduced TCR signaling upon stimulation with α-galactosylceramide, suggesting that Kidins220 promotes TCR signaling in peripheral iNKT cells. Thus, Kidins220 reduces or promotes signaling dependent on the iNKT cell developmental stage.

## Teaser

The scaffold protein Kidins220 regulates T cell receptor (TCR) signal strength in invariant natural killer T (iNKT) cells.

## INTRODUCTION

T lymphocytes are a crucial part of the adaptive immune system. αβ T cells, which express the αβ T cell antigen receptor (TCR) to recognize foreign antigens, are divided into conventional T cells, such as CD4^+^ T helper and CD8^+^ T killer cells, and unconventional T cells, such as invariant natural killer T (iNKT) and mucosal-associated invariant T (MAIT) cells. The αβ TCR comprises the TCRα and TCRβ chains, which bind to the antigen, and the CD3 subunits, which transduce the signal of antigen binding into the cell (*1*). Signaling by the TCR steers T cell development in the thymus and T cell activation in the periphery.

Development in the thymus starts with CD4^−^CD8^−^ (DN) thymocytes, in which the TCRβ gene locus gets rearranged to allow expression of a TCRβ chain (*2*). Subsequently, the thymocytes up-regulate CD4 and CD8 developing into double-positive (DP) cells, which rearrange the TCRα gene locus. Following successful TCRα recombination, DP thymocytes develop into conventional T cells and need to be positively selected by binding to self-peptides presented by major histocompatibility complex (MHC) on the surface of thymic epithelial cells (*3*–*9*). If the TCR binds too strongly to peptide-MHC, the T cells will be negatively selected and eliminated from the repertoire to prevent autoimmunity. If the TCR binds too weakly, the cells die by neglect (*10*). CD5 expression levels reflect TCR signaling strength during positive selection (*11*, *12*). The stronger the signal is, the more CD5 is expressed, most likely to mitigate the TCR signal because CD5 is an inhibitory receptor (*12*). Upon positive selection, thymocytes develop into single-positive (SP) CD4^+^ or CD8^+^ T cells and migrate to the periphery.

Unlike conventional T cells, iNKT cells react on threats almost immediately by the secretion of cytokines, such as interleukin-4 (IL-4), IL-17, and inteferon-γ (IFN-γ) (*13*–*20*). Hence, iNKT cells are crucial for initiating and regulating immune responses. With their invariant TCR, they recognize a variety of glycolipids, such as α-glucosyldiacylglycerol, which originates from the cell wall of several bacteria. These glycolipids are presented by CD1d, an MHC I–like molecule, on antigen-presenting cells (APCs) (*21*).

To become positively selected in the thymus to the iNKT lineage, DP cells carrying the V_α_14J_α_18 TCR (in mice) bind to glycolipid-loaded CD1d expressed on DP thymocytes (*22*–*24*). These glycolipids are self-molecules (*25*). This binding generates a strong TCR signal that is required for initial iNKT cell selection (*26*, *27*). Subsequently, iNKT cells develop into terminally differentiated effector cells in the thymus, such as iNKT1 (secreting IFN-γ), iNKT2 (secreting IL-4), or iNKT17 (secreting IL-17) cells (*13*).

Directly after their selection into the iNKT lineage, the developing iNKT cells are CD24^+^ CD44^−^ NK1.1^−^ and called stage 0 or iNKT0 cells (*28*). Initially, a linear model was proposed in which the cells would develop through stage 1 (CD24^−^ CD44^−^ NK1.1^−^), stage 2 (CD24^−^ CD44^+^ NK1.1^−^), and stage 3 (CD24^−^ CD44^+^ NK1.1^+^) cells (*29*). More recent experiments including lineage tracing via single-cell RNA sequencing (scRNA-seq) have revealed branched progression through development (*27*, *30*–*33*). iNKT0 cells develop into a precursor state, called iNKTp. From there they progress either to iNKT2 cells, which are very similar to the iNKTp cells, or to iNKT17 cells (both are in stage 2). The iNKT2 cells then further develop into iNKT1 cells (stage 3 cells) (*27*, *33*, *34*). The most immature iNKT1 cells are called iNKT1a and differentiate via the iNKT1b state to the iNKT1c cells with effector signatures.

The exact developmental route taken depends on the iNKT cell’s TCR signal strength (*35*). Medium and strong TCR signals result in the preferential generation of iNKT2 and iNKT17 cells, respectively. In contrast, weak signals promote the development of iNKT1 cells. Thus, genetic mutations reducing TCR signal strength, such as those in ZAP-70 (*36*, *37*), were shown to decrease iNKT2 and iNKT17 cell numbers, whereas iNKT1 cell numbers were increased (*35*).

We previously identified the scaffold protein kinase D–interacting substrate of 220 kDa (Kidins220), also called ankyrin repeat–rich membrane spanning, to bind to the antigen receptors, namely, the TCR and the B cell receptor (*38*, *39*). Kidins220 is a large protein with four transmembrane regions with both termini reaching into the cytoplasm. It has various protein-protein interaction domains—such as 11 ankyrin repeats, a SAM domain, a proline-rich sequence, and a PDZ-binding motif—and thus is thought to act as a signaling hub (*40*, *41*). It was initially found in neurons, where it interacts with the nerve growth factor receptor and neurotrophin receptors (*42*–*44*). We have studied the function of Kidins220 in a murine αβ T cell line by using a short hair RNA–based Kidins220 knockdown (KD) (*38*). Following TCR stimulation, downstream signaling was reduced in Kidins220 KD cells, although TCR levels on the surface were increased. After TCR stimulation, Kidins220 KD cells were less activated, as seen by reduced production of IL-2, IFN-γ, and CD69. Hence, Kidins220 was described as an important positive regulator of TCR signaling in vitro (*38*).

Here, we gained insight into the function of Kidins220 in T cells in vivo. To this end, we generated T cell–specific Kidins220 KO (T-KO) mice, analyzed T cells, and focused on the iNKT subset.

## RESULTS

### Kidins220 promotes the development of CD4 and CD8 SP thymocytes

**Fig. 1.**
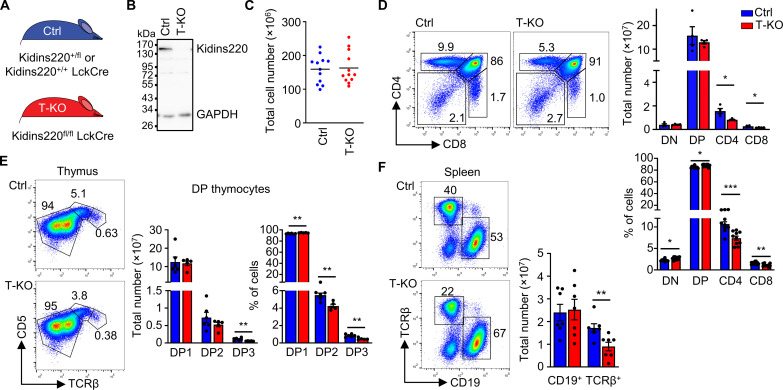
Development of SP thymocytes is reduced in Kidins220 T-KO mice. (**A**) Genotypes of Ctrl and T cell–specific Kidins220 KO (T-KO) mice are shown. (**B**) Thymocytes of Ctrl and T-KO mice were lysed, and proteins were analyzed by SDS–polyacrylamide gel electrophoresis and anti-Kidins220 and anti–glyceraldehyde-3-phosphate dehydrogenase (GAPDH) Western blotting. (**C**) Total thymocyte numbers of Ctrl and T-KO are depicted (*n* = 12). (**D**) Thymocyte development of Ctrl and T-KO mice was analyzed using anti-CD4 and anti-CD8 antibodies. Total cell numbers and percentage of cells are shown (cell number, *n* = 4; and percent, *n* = 10). (**E**) DP cells were analyzed using anti-TCRβ and anti-CD5 antibodies (DP1: TCRβ^low^CD5^low^; DP2: TCRβ^int^CD5^high^; DP3: TCRβ^high^CD5^int^). Graphs show total cell numbers and percentage of cells (*n* = 6). (**F**) B and T cells in the spleen of Ctrl and T-KO mice were analyzed by anti-CD19 and anti-TCRβ staining. Total cell numbers are shown (*n* = 7 to 10). For all figures, information on the statistics is given in Materials and Methods.

To analyze the role of Kidins220 in T cell development, we generated a T-KO by crossing Kidins220 floxed (Kidins220^fl/fl^) (*45*, *46*) with LckCre mice (*47*) (Fig. 1A). In the latter, the Cre recombinase is expressed under the proximal *Lck* promoter very early in T cell development, starting at the DN2 stage (*47*, *48*). Western blot analysis confirmed successful deletion of Kidins220 in T cells (Fig. 1B). The total number of thymocytes were similar in T-KO and Ctrl mice (Fig. 1C). Number and percentage of cells in both CD4^+^ and CD8^+^ SP stages were reduced in T-KO thymi compared to the Ctrl ones. Concomitantly, the percentages of DN and DP cells were slightly increased (Fig. 1D). The ratio of CD4^+^ to CD8^+^ SP cells remained unchanged (fig. S1A), indicating that CD4^+^ and CD8^+^ cells were equally affected.

As the proximal *LckCre* is used in T-KO mice to delete the floxed Kidins220 gene and is hardly expressed in γδ T cells (*48*), total numbers of γδ T cells were not affected in T-KO thymi (fig. S1B).

A reduction of SP cell numbers could be due to inefficient positive selection. To analyze this, we determined different stages of the selection process according to the expression levels of TCRβ and CD5 in DP cells (*49*, *50*). Preselection DP1 (CD5^low^TCRβ^low^) thymocytes were equally abundant in Ctrl and T-KO (Fig. 1E). DP2 (CD5^high^TCRβ^int^) cells, the recently (12 to 48 hours) positively selected thymocytes and were percentwise reduced and showed a tendency to be slightly reduced in numbers in the T-KO. Percentwise they were reduced (Fig. 1E). This suggests that there might be a slight reduction in positive selection in the absence of Kidins220. Strikingly, DP3 (CD5^int^TCRβ^high^) cells, which consist of the positively selected cells (~60 hours) (*49*), were reduced in numbers and in percent in the T-KO. This result indicates that after positive selection, those cells might be lost over time in the absence of Kidins220. Similar results were obtained when cells were analyzed according to CD69 and TCRβ levels (fig. S1C).

Reduced production of SP thymocytes could cause less T cells to be present in the periphery. Splenic T cell numbers were diminished twofold in T-KO compared to Ctrl mice. As expected, B cell numbers were unaffected in T-KO mice (Fig. 1F and fig. S1D).

### Absence of Kidins220 reduces iNKT cell numbers

Besides conventional T cells, unconventional T cells are lately emerging as being more abundant and influential than previously recognized. Unconventional T cells include MAIT cells, iNKT, and γδ T cells and often make up the majority of T cells in tissues. Therefore, we tested whether unconventional T cell numbers were also affected by the loss of Kidins220.

**Fig. 2.**
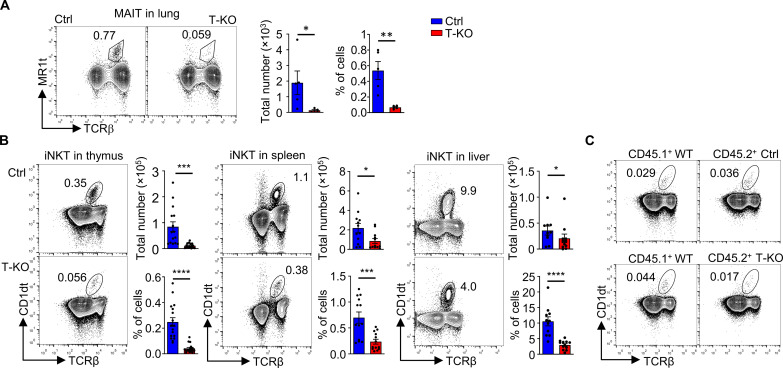
The numbers of unconventional T cells are decreased in T-KO mice. (**A**) Single-cell suspension of lymphocytes isolated from lung were stained with MR1 tetramers (MR1t) and anti-TCRβ antibodies to identify the MAIT cells (*n* = 5). Total cell numbers and relative abundance among lymphocytes are depicted. (**B**) iNKT cells were analyzed by staining lymphocytes from Ctrl and T-KO thymus, spleen, and liver with CD1d tetramers (CD1dt) and anti-TCRβ antibodies (*n* > 11). (A and B) Total and relative cell numbers are depicted. (**C**) Flow cytometric analyses of iNKT cells in the thymus of mixed bone marrow chimeric mice are shown. RAG2 KO mice were lethally irradiated and reconstituted with bone marrow cells from CD45.1^+^ WT and CD45.2^+^ Ctrl or CD45.2^+^ T-KO mice in a 1:1 ratio. A representative analysis of the thymocytes of the WT/Ctrl (top) and WT/T-KO (bottom) chimeras is shown (*n* = 7 to 8).

MAIT cells in the lung were identified by staining with anti-TCRβ antibodies and 5-OP-RU [5-(2-oxopropylideneamino)-6-d-ribitylaminouracil]–loaded MR1 tetramers. MR1 molecules, which are an MHC class I homolog, loaded with the vitamin B intermediate 5-OP-RU bind to the invariant TCR of MAIT cells (*51*–*53*). The number of MAIT cells was reduced in T-KO mice compared to Ctrl mice (Fig. 2A). With a reduction of 13-fold in numbers, the phenotype was stronger than the one of conventional T cells.

Next, we analyzed iNKT cells by staining with CD1d tetramers loaded with PBS57, an analog of α-galactosylceramide (αGalCer). When bound to CD1d, αGalCer (and PBS57) serves as potent ligand for the iNKT cell’s invariant TCR (*54*, *55*). In the thymus of T-KO mice, iNKT cell numbers were reduced sixfold, and in peripheral organs such as spleen and liver, iNKT cells were reduced three- and twofold, respectively (Fig. 2B). These findings suggested that iNKT cell development is hampered in the absence of Kidins220.

During thymic development, iNKT precursor cells are selected by DP thymocytes presenting glycolipid-loaded CD1d molecules (*15*, *23*). We found that CD1d levels were reduced by 1.2-fold on DP thymocytes of T-KO mice compared to Ctrl mice (fig. S2A). CD4 and CD8 levels showed no differences, indicating that there was not a general reduction in surface protein levels (fig. S2B). The lower CD1d expression on DP cells could have caused a reduced selection of iNKT cells because the latter need strong TCR signals for positive selection (*26*).

To test this hypothesis, we generated bone marrow chimeras, for which equal amounts of bone marrow cells from CD45.1^+^ wild-type (WT) mice were mixed with CD45.2^+^ Ctrl or CD45.2^+^ T-KO bone marrow cells and injected intravenously into irradiated C57BL/6 Rag2^−/−^ (Rag2 KO) mice. In this setup, CD45.2^+^ T-KO iNKT precursor cells have access to normal amounts of CD1d molecules on the surface of CD45.1^+^ WT DP thymocytes. After 8 weeks, we analyzed iNKT cells in the recipient thymi. When WT cells were co-injected with Ctrl cells, the percentages of iNKT cells were similar in the WT CD45.1^+^ and Ctrl CD45.2^+^ populations (Fig. 2C, top). However, after reconstitution with CD45.1^+^ WT and CD45.2^+^ T-KO bone marrow cells, the thymic T-KO iNKT cells showed a reduction of about twofold compared to the WT cells (Fig. 2C, bottom and fig. S2C). Hence, the decrease of iNKT cell numbers in T-KO mice was an iNKT intrinsic effect because it could not be rescued once T-KO cells had access to WT amounts of CD1d during development.

Concerning the reduced numbers of MAIT cells, we found that MR1 levels were not changed between T-KO and Ctrl DP thymocytes (fig. S2D). Hence, the reduction of MAIT cells was most likely also due to intrinsic effects.

### Kidins220 enhances T cell activation and TCR signaling in splenic iNKT cells

**Fig. 3.**
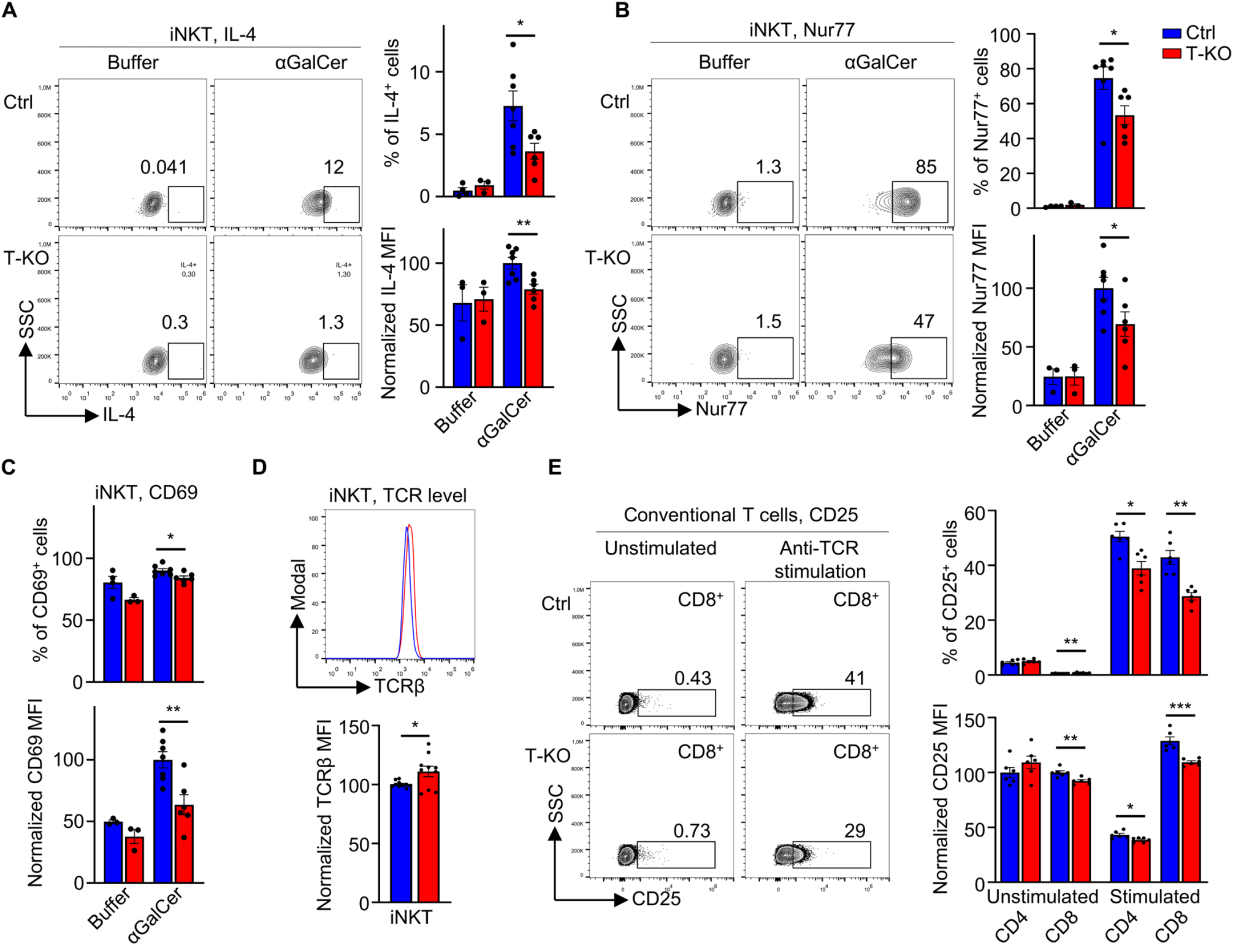
Reduced activation of iNKT cells from T-KO mice after αGalCer challenge. (**A** and **B**) Ctrl and T-KO mice were euthanized 2 hours after intraperitoneal injection of αGalCer or buffer alone. Expression of IL-4 (A) and Nur77 (B) was analyzed in splenic iNKT cells by intracellular flow cytometry using anti–IL-4 and anti-Nur77 antibodies (*n* = 6). Plots were pregated on CD1dt^+^ TCRβ^+^ iNKT cells and MFI values were normalized to the ones of Ctrl samples for each independent experiment. (**C**) Statistics of the percent of CD69-expressing iNKT cells and of the CD69 MFI is shown after flow cytometry using anti-CD69 antibodies (*n* = 6). (**D**) TCRβ expression levels on splenic iNKT cells from Ctrl and T-KO mice were determined by flow cytometry (*n* = 10). (**E**) Splenic T cells from Ctrl and T-KO mice were stimulated for 4 hours using anti-CD3 and anti-TCRβ antibodies. Cells were then stained with anti-CD4, anti-CD8, and anti-CD25 antibodies and analyzed by flow cytometry (*n* = 6).

Next, we analyzed whether Kidins220 affects iNKT cell effector functions in the periphery. Because iNKT cells are a major source of early IL-4 and IFN-γ production after αGalCer challenge, we analyzed the production of these cytokines by splenic iNKT cells 2 hours after intraperitoneal αGalCer injection. By intracellular flow cytometric staining, we found that the percentage of iNKT cells producing IL-4 or IFN-γ were reduced in T-KO compared to Ctrl mice (Fig. 3A and fig. S3A). Further, the mean fluorescence intensity (MFI) for both cytokines was reduced in the cytokine-producing cells from T-KO mice, indicating that each cell produced less. Injection of buffer alone did not lead to detectable cytokine production. Thus, T-KO mice contained less activated effector iNKT cells compared to Ctrl mice upon in vivo TCR stimulation. This is in line with reduced TCR-mediated T cell activation in a conventional T cell line in which Kidins220 expression was down-regulated (*38*).

Less production of IL-4 and IFN-γ could be due to reduced signaling via the iNKT cells’ TCR. Nur77 is expressed after TCR engagement, thus reporting on TCR signal strength (*26*, *56*, *57*). We found that αGalCer-treated T-KO mice had 60%, whereas Ctrl mice had 81% Nur77^+^ iNKT cells in the spleen (Fig. 3B), and that the Nur77 expression levels in iNKT cells had the tendency of being lower in T-KO compared to Ctrl. This indicates that in splenic iNKT cells, the absence of Kidins220 reduces TCR signal strength. Diminished TCR signaling in T-KO iNKT cells would also explain the reduced CD69 expression levels following αGalCer challenge in vivo (Fig. 3C and fig. S3B).

Splenic T-KO iNKT cells expressed 1.1-fold higher TCR levels compared to Ctrl iNKT cells as detected by an anti-TCRβ stain (Fig. 3D). This is consistent with findings that a down-regulation of Kidins220 in a mature conventional T cell line led to slightly increased TCR levels (*38*) and excludes that less TCR signaling in the absence of Kidins220 was due to lower TCR levels.

To test whether conventional T-KO peripheral T cells were also less activated, we stimulated splenic CD4^+^ and CD8^+^ T cells ex vivo for 4 hours with anti-TCR antibodies. We found that these T cells expressed the high affinity IL-2 receptor (CD25) activation marker less when Kidins220 was missing (Fig. 3E and fig. S3C). The unstimulated CD4^+^ cells showed some CD25 on their surface, being in line with the expression of CD25 in regulatory T cells which are CD4-positive. In conclusion, Kidins220 promotes TCR signaling in peripheral iNKT and conventional T cells.

### Development of T-KO iNKT cells is impeded in T-KO mice, with strongly reduced iNKT1 numbers

**Fig. 4.**
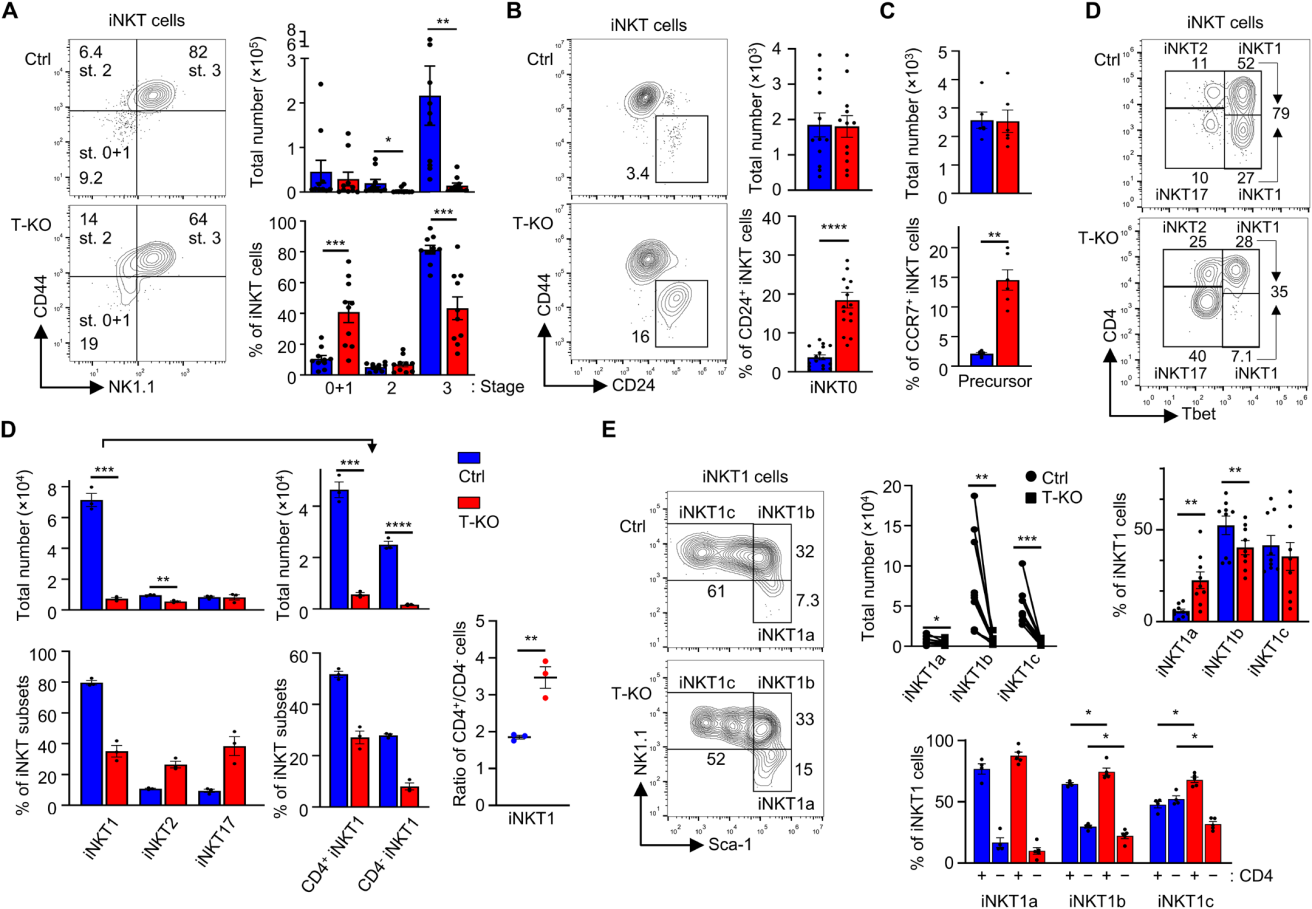
Partial block of iNKT development from stage 2 to stage 3 in T-KO. (**A**) Thymic CD1dt^+^ TCRβ^+^ iNKT cells were analyzed by flow cytometry using anti-CD44 and anti-NK1.1 antibodies and grouped into stages 0 + 1, 2, and 3. Graphs show total iNKT and relative cell numbers (*n* = 10). (**B**) The amount of CD24^+^ iNKT0 cells was analyzed using anti-CD24 and anti-CD44 antibodies (*n* = 12 to 16). (**C**) Precursor iNKT cells were identified by their expression of CCR7^+^. The flow cytometry plots are shown in fig. S4B (*n* = 6). (**D**) CD1dt^+^ TCRβ^+^ thymic iNKT cells were stained with anti-CD4 and with anti-Tbet antibodies intranuclearly and analyzed by flow cytometry (*n* = 3). (**E**) Thymic iNKT1 cells were identified as above and subdivided into iNKT1a, iNKT1b, and iNKT1c cells by using anti–Sca-1 and anti-NK1.1 antibodies (*n* = 11). Subsequently, each subset was divided into CD4^+^ and CD4^−^ populations based on anti-CD4 antibody staining (*n* = 4). In all panels, cells were pregated for CD1dt^+^ TCRβ^+^ iNKT cells.

Reduced iNKT cell numbers in T-KO mice might originate from an inefficient development of these cells in the thymus. To elucidate iNKT cell development, we analyzed thymic iNKT cells (CD1dt^+^ TCRβ^+^) from Ctrl and T-KO mice by flow cytometry. These cells were divided into the established stages 0 and 1 (stage 0 + 1; CD44^−^ NK1.1^−^), stage 2 (CD44^+^ NK1.1^−^), and stage 3 (CD44^+^ NK1.1^+^; Fig. 4A). Further, stage 0 was assessed separately (CD44^−^ CD24^+^; Fig. 4B). Similar total iNKT cell numbers in stages 0 and 1 together and in stage 0 alone were detected in T-KO and Ctrl mice. In sharp contrast, stage 2 and 3 cell numbers were reduced in T-KO mice, with stage 3 cells being affected the most (Fig. 4A). This reduction led to a percentwise increase of cells in stages 0 and 1 and a decrease in stage 3. Accordingly, the ratio of T-KO to Ctrl cells is much lower in stage 3 compared to stage 0 (fig. S4A). Furthermore, CCR7^+^ iNKT precursor cells (*31*) were equal in numbers in Ctrl and T-KO mice (Fig. 4C and fig. S4B).

The strong reduction of stage 3 iNKT cell numbers suggested that IFNγ-producing iNKT1 cell numbers were reduced already in the thymus. The iNKT1 cells express the transcription factor Tbet and are either positive or negative for CD4. Instead, iNKT2 cells are Tbet^−^ CD4^+^, whereas iNKT17 cells are Tbet^−^ and mostly CD4^−^ (*13*). Numbers of iNKT1 cells were reduced in T-KO mice by 10-fold, iNKT2 cell numbers by twofold, while the CD4^−^ iNKT17 cell numbers were unchanged (Fig. 4D). This shows that the absence of Kidins220 strongly interferes with iNKT1, slightly with iNKT2 and not with CD4^−^ iNKT17 development. Numbers of both CD4^+^ and CD4^−^ iNKT1 cells were reduced, with CD4^−^ iNKT1 cells being affected stronger (Fig. 4D). Using CD122 as an alternative strategy to identify iNKT1 cells (*58*), we confirmed the strong reduction of those cells in the absence of Kidins220 (fig. S4C).

Because iNKT1 (NK1.1^+^ CD44^+^) cells showed the strongest reduction, we further studied them by subdividing them into iNKT1a, iNKT1b, and iNKT1c subsets based on Sca-1 and NK1.1 expression (*33*). iNKT1a cell numbers were only slightly reduced in T-KO mice. (Fig. 4E). However, there was a strong reduction of iNKT1b and iNKT1c cell numbers, indicating that Kidins220 is mostly required in those subsets.

The ratio of CD4^+^ to CD4^−^ iNKT1 cells was larger in T-KO mice (Fig. 4D). In Ctrl mice, there were four times and two times more CD4^+^ than CD4^−^ iNKT1a and iNKT1b cells, respectively (Fig. 4E). This was unchanged in T-KO mice. In Ctrl mice CD4^−^ cells seem to “catch up” at the iNKT1c cell subset, because there were equal numbers of CD4^+^ and CD4^−^ cells. However, in T-KO mice, there were still more CD4^+^ than CD4^−^ iNKT1c cells. Thus, the loss of Kidins220 interferes with the later stages of iNKT1 development and hinders CD4^−^ iNKT1c cells to develop.

### T-KO stage 3 iNKT cells are more susceptible to apoptosis

**Fig. 5.**
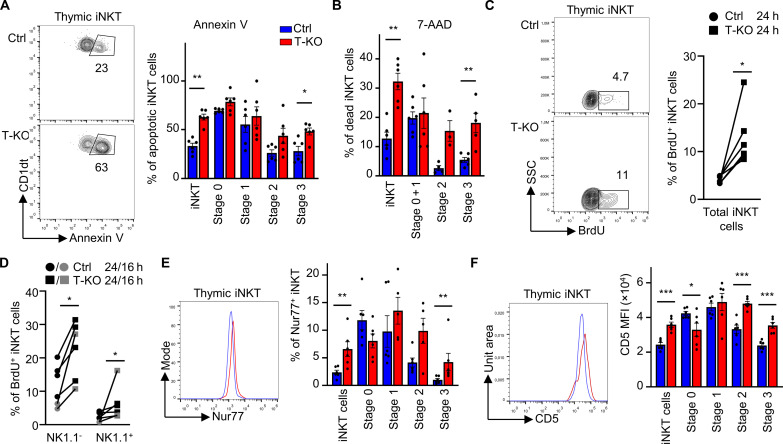
Stage 3 thymic iNKT cells of T-KO mice are more susceptible to apoptosis and exhibit stronger TCR signaling than the ones of the Ctrl. (**A**) To quantify apoptosis in iNKT cells, thymocytes were cultivated for 18 hours in RPMI medium supplemented with 10% FBS. Thymocytes were stained with annexin V to detect apoptotic cells. Cells were pregated on CD1dt^+^ TCRβ^+^ iNKT cells. iNKT stages were analyzed using anti-CD24, anti-CD44, and anti-NK1.1 antibodies as in Fig. 4. Dead cells based on FSC, and SSC were excluded. The graph depicts the percentage of annexin V^+^ cells in the indicated iNKT stages (*n* = 6). (**B**) Dead thymocytes were detected using 7-AAD by flow cytometric analysis and antibodies to gate on the stages given. Graph shows percentage of 7-AAD–positive cells in the indicated iNKT stages (*n* = 6). (**C** and **D**) Ctrl and T-KO mice were intraperitoneally injected with BrdU to analyze proliferating cells. BrdU-treated mice were euthanized 16 (gray) or 24 hours (black) after BrdU injection, and thymocytes were stained with anti-BrdU antibodies. CD1dt^+^ TCRβ^+^ iNKT cells and iNKT cells subdivided into NK1.1^+^ and NK1.1^−^ cells were analyzed using flow cytometry. Graphs show relative numbers of BrdU^+^ cells (*n* = 6 to 7). (**E**) Thymic iNKT cells and iNKT stages were analyzed using anti-Nur77 antibodies. Cells were pregated on CD1dt^+^ TCRβ^+^ iNKT cells, and iNKT stages were identified as above. Graphs show relative numbers of Nur77^+^ iNKT cells (*n* = 6). (**F**) CD5 MFI in thymic iNKT cells was assessed by flow cytometry using anti-CD5 antibodies. The iNKT cells and stages were identified as above (*n* = 6).

In the absence of Kidins220, iNKT cell numbers in the thymus were increasingly reduced the further they progressed in development. This reduction might be caused by an increase in apoptosis or reduced proliferation. We first tested whether iNKT T-KO cells exhibit increased apoptosis. To this end, thymocytes were cultured overnight ex vivo and subsequently stained with annexin V to identify apoptotic cells and with antibodies to gate on the different developmental stages as above. In general, we detected increased ratios of apoptotic cells among iNKT T-KO thymocytes. Especially, stage 3 cells contained statistically more apoptotic cells in the T-KO compared to the Ctrl (Fig. 5A). Overall, there was a tendency that the further iNKT cells progressed in development, the more apoptotic cells were present in the T-KO compared to the Ctrl (Fig. 5). As seen by 7-Aminoactinomycin D (7-AAD) staining, thymic stage 3 T-KO iNKT cells also contained higher percentages of dead cells than the stage 3 cells of Ctrl mice (Fig. 5B and fig. S5A). In stages 0 and 1 gated together, no difference between T-KO and Ctrl was seen. These findings were further supported by visualization of apoptotic cells based on active Caspases 3 and 7 staining (fig. S5B). In line with the strongest reduction of NK1.1^+^ iNKT cell numbers (i.e., stage 3 numbers) in T-KO mice (Fig. 4A), the increase of apoptosis (T-KO versus Ctrl) was stronger in NK1.1^+^ iNKT cells compared to the one of NK1.1^-^ iNKT cells (fig. S5B).

The anti-apoptotic protein BCL_XL_ is important for cell survival during iNKT cell development (*59*). BCL_XL_ was expressed to higher level in the T-KO iNKT cells compared to the Ctrl (fig. S5C). Thus, those cells that survived might have been the ones where BCL_XL_ was present at higher levels. BCL2 was also shown to support iNKT survival (*60*), but in our experiments, BCL2 was expressed similarly in T-KO and Ctrl cells.

Next, we tested for iNKT cell proliferation in vivo by injecting 5-bromo-2′-deoxyuridine (BrdU) intrapeitoneally into T-KO and Ctrl mice. Because BrdU incorporates into the DNA during DNA synthesis, the proliferated cells can be visualized by flow cytometry using anti-BrdU antibodies. Unexpectedly, T-KO iNKT cells had proliferated more compared to the Ctrl cells (Fig. 5C and fig. S5D). This held true for NK1.1^−^ and NK1.1^+^ iNKT cells (Fig. 5D).

In conclusion, Kidins220-deficient thymic iNKT cells not only proliferated more but also were more prone to apoptosis than the Ctrl cells. The latter might explain the reduction of iNKT numbers in T-KO mice.

### TCR signaling is elevated in stage 3 T-KO iNKT cells

Enhanced proliferation and apoptosis in thymic T-KO iNKT cells could be caused by stronger TCR signaling during development. As Nur77 expression levels can be used to quantify TCR signal strength (*26*), we analyzed the percentage of Nur77^+^ iNKT cells in the thymus. In the total iNKT population, there were three times more Nur77^+^ iNKT cells in T-KO mice compared to Ctrl mice (Fig. 5E). The percent of Nur77^+^ cells in the different iNKT stages is differentially affected by the absence of Kidins220. At the early stages (stages 0 and 1), there were no statistically significant differences between Ctrl and T-KO. In stage 2, there was a tendency for more Nur77^+^ cells in the T-KO. Last, in stage 3, a large increase of Nur77^+^ cells in the T-KO was found (namely, fourfold compared to Ctrl). Nur77 expression levels in the cells as quantified by the MFI revealed the same trend (fig. S5E).

In addition, 1.4-fold elevated CD5 levels, also indicating enhanced TCR signal strength, were found in total thymic iNKT cells from T-KO compared to Ctrl mice (Fig. 5F). Consolidating the Nur77 data, we detected equal CD5 levels in stages 0 and 1 and enhanced levels in stages 2 and 3. This was not the case in stages 0 and 1. Elevated CD5 expression was preserved in peripheral NK1.1^+^ and NK1.1^−^ iNKT cells from the spleen (fig. S5F).

Because we found slightly enhanced TCR expression levels in splenic iNKT cells (Fig. 3D), we stained thymic iNKT cells with anti-TCRβ antibodies or CD1dt (fig. S2, G and H). As expected, we detected slightly higher TCR levels in stages 2 and 3 comparing the T-KO with the Ctrl. In stages 0 and 1, the CD1dt staining was equal.

Strikingly, these data suggest that Kidins220 specifically reduces TCR signaling at stages 2 and 3 of iNKT development, but not at stages 0 and 1.

### scRNA-seq reveals that Aiolos is down-regulated in iNKT T-KO compared to Ctrl cells

**Fig. 6.**
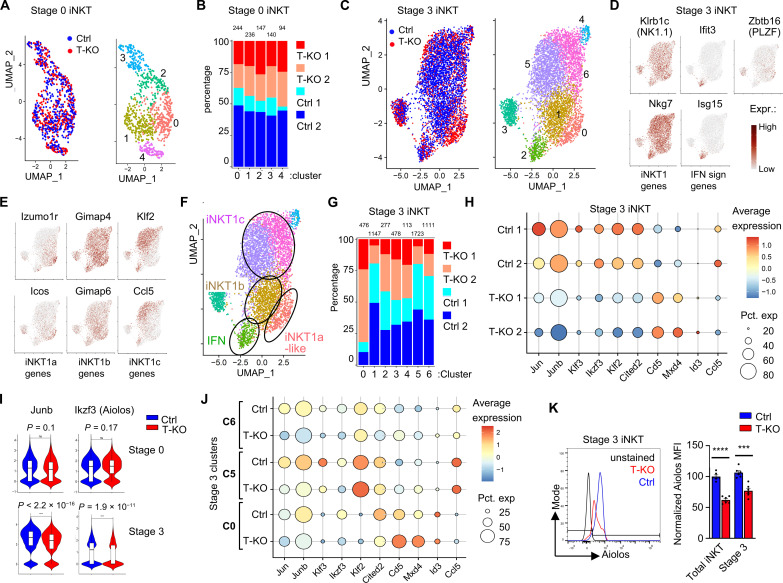
scRNA-seq reveals differences between stage 3 T-KO and Ctrl cells. (**A**) UMAP plot of two stage 0 iNKT scRNA-seq datasets of Ctrl and T-KO were integrated, and cells were colored by their Ctrl or T-KO origin (left) or by inferred cluster identity (right). (**B**) Relative abundance of T-KO cells and Ctrl cells (from each replica) in the stage 0 clusters is given. Total number of cells in each cluster is given at the top. (**C**) UMAP plot of two stage 3 iNKT scRNA-seq datasets of Ctrl and T-KO was integrated, and cells were colored by origin (left) or by inferred cluster identity (right). (**D** and **E**) Expression of two typical genes used to define iNKT1, interferon signaling, iNKT1a, iNKT1b, and iNKT1c cells. (**F**) Identification of the cell clusters according to the expression of marker genes. (**G**) Relative abundance of T-KO cells and Ctrl cells (from each replica) in the stage 3 clusters is given. (**H**) Dot plot showing expression of 10 genes differentially expressed in stage 3 Ctrl and T-KO cells. Color represents the scaled expression of the gene in the respective condition, and dot size represents the fraction of cells in the condition expressing the gene. (**I**) Violin plots included with box plots show the normalized transcript counts of *Junb* and *Ikzf3* in stage 0 and stage 3 cells from Ctrl and T-KO. Significance was assessed using Wilcoxon test. (**J**) Dot plot showing scaled expression of 10 genes in stage 3 clusters C0, C5, and C6 for Ctrl and T-KO. (**K**) Ctrl and T-KO thymocytes were stained with an anti-Aiolos antibody and measured by flow cytometry. Total iNKT and stage 3 cells were gated as in Fig. 4 and analyzed (*n* = 6).

One notable effect of the absence of Kidins220 was the strong reduction of iNKT cell numbers at stage 3 but not at stage 0 of development (Fig. 4, A and B). Thus, we aimed to uncover which genes are regulated by Kidins220 in these stages and thus to obtain an insight into Kidins220’s function. To this end, we isolated stage 0 and stage 3 iNKT cells of Ctrl and T-KO mice and determined their transcriptomic profiles by droplet-based scRNA-seq using 10X Genomics (scRNA-seq; fig. S6A). This was done twice in parallel (Ctrl 1, Ctrl2 and T-KO 1, T-KO 2). After the filtering steps (fig. S6A), 861 stage 0 cells were considered for our analysis. We used uniform manifold approximation and projection (UMAP) for dimensionality reduction and applied unsupervised cluster analysis using the Seurat algorithm (*61*). This yielded five distinct clusters (Fig. 6A), suggesting a previously unappreciated transcriptional heterogeneity of stage 0 iNKT cells. Because the stage 0 Ctrl and T-KO cells were very similarly distributed in the UMAP (Fig. 6, A and B), we did not follow the different stage 0 clusters. There were only three differentially expressed genes (*Hspa1b*, *Resf1*, and *2410006H16Rik*; table S1), and this is in line with our flow cytometry analyses in which we hardly saw differences between Ctrl and T-KO stage 0 cells.

When analyzing the 5325 stage 3 cells, we found that the Ctrl cells (blue) are localized at different positions in the UMAP plot as the T-KO cells (red, Fig. 6C). This is reflected in 83 differentially expressed genes (table S2). The cells were grouped into seven different clusters (C0 to C6; Fig. 6C). To determine the identity of our clusters, we used genes differentially expressed in different iNKT1 subsets as identified by previous transcriptomic analyses (*27**,* *32**,* *33**,* *62**,* *63*). Expression of *Klrb1c* (NK1.1), *Nkg7*, *Tbx21* (Tbet), and *Il2rb* confirms that these stage 3 cells are the iNKT1 cells (Fig. 6D and fig. S6B). Marker genes *Ifit3*, *Isg15*, *Stat1*, and *Irf7* show that C2 corresponds to iNKT cells with a type I IFN signature. Gradual loss of *Zbtb16* (PLZF) from C0 to C6 (Fig. 6D) indicates that the iNKT1 cells develop from C0 to C6 (*33**,* *64*). Presence of *Izumo1r* and *Icos* and lack of *S1006a* and *Ifng* (Fig. 6E and fig. S6B) identified C0 as the very early iNKT1 cells, similar to iNKT1a cells (*32*, *33*). Because iNKTa cells are identified by being NK1.1^−^, but our cells contain NK1.1 (Fig. 6D), we think that C0 cells are very late iNKT1a cells that are about to up-regulate NK1.1, starting to do the developmental step to iNKT1b. Here, we call them iNKT1a-like. iNKT1b cells are enriched in *Gimap4* and *Gimap6*, and this is the case in C1 (Fig. 6E and fig. S6B).

C5 and C6 are enriched in *Klf2*, *Ccl5*, *Ifng*, *Gzma*, *Klra7*, *Klra9*, and *Fcer1g*, suggesting that these are the iNKT1c cells having effector function. The trajectory analysis (fig. S6C) confirms the assignment of the iNKT1 subsets to the clusters (Fig. 6F). Identity of C3 and C4 remains unclear, and they are not enriched in cycling cells.

Examining the individual stage 3 clusters, we found that T-KO cells dominate C0 (iNKT1a-like) and are reduced in C1, C5, and C6 (iNKT1b and iNKT1c; Fig. 6G). These data suggest that there is a partial block in the development from iNKT1a to iNKT1b in the T-KO mice. This perfectly substantiates our previous phenotyping analysis (Fig. 4E) and could be caused by enhanced apoptosis the further the cells develop.

Next, we examined the differential expressed genes that were significantly up or down-regulated in the T-KO compared to the Ctrl in stage 3 cells (Fig. 6H and supplemental table S2). Of particular interest were genes encoding for transcription factors. *Mxd4* and *Id3* were up-regulated, whereas *Jun*, *Junb*, *Klf3*, *Ikzf3*, *Klf2*, *Cited2*, *Fos*, *Phf1* and *Klf6* were down-regulated in T-KO cells (Fig. 6, H and I, and fig. S6D). None of these transcripts was differentially expressed at stage 0 (fig. S6E). As a control, the *Cd5* transcript was up-regulated in the T-KO at stage 3 (Fig. 6H), but not at stage 0 (fig. S6, D and E), being in line with the protein data (Fig. 5E).

When comparing Ctrl with T-KO, only some of the genes were differentially expressed in the stage 3 clusters C0, C1, C5, or C6 (Fig. 6J and fig. S7)—the clusters where Ctrl and T-KO cells were not equally present. For example, *Klf2* and *Ccl5* were very similar within each cluster, but *Junb*, *KLf3*, *Ikfz3*, and *Cited2* were strongly expressed in the Ctrl. Important to get insight into the function of Kidins220 is a detailed analysis of cluster C0, since in C0, the T-KO cells have a partial block in development. *Mxd4* was up-regulated in the T-KO, and *Junb*, *Cited2*, and *Ikfz3* (encoding for the protein Aiolos) were down-regulated (Fig. 6J and fig. S7). Being downstream of Kidins220, they might be involved in Kidins220 function.

Aiolos is a member of the Ikaros family characterized by zinc finger domains implicated in DNA binding and protein interactions (*65*). Its function in B cell development has been described (*66*), but its role in iNKT cell development has not been studied so far. Thus, we focused on Aiolos in the remainder of this study. Staining for the protein shows that it is 1.4-fold less expressed in the T-KO compared to the Ctrl at stage 3 (Fig. 6K), confirming the scRNA-seq data. That Kidins220 controls the expression of Aiolos is specific to iNKT cells because Aiolos levels in DP and SP thymocytes are the same in Ctrl and T-KO mice (fig. S6F).

### The Aiolos KO phenocopies the Kidins220 T-KO

**Fig. 7.**
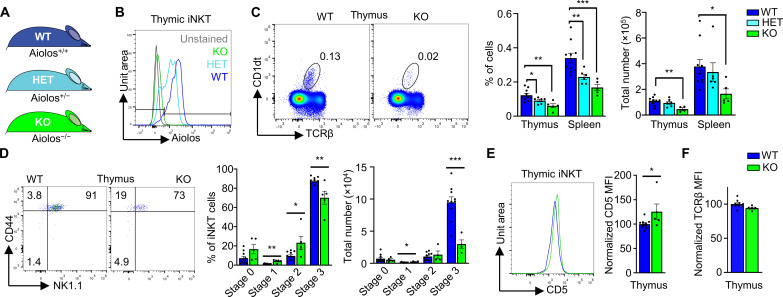
iNKT cells of Aiolos KO mice resemble those of the Kidins220 T-KO mice. (**A**) Genotypes of WT, heterozygous (HET), and homozygous (KO) Aiolos KO mice are shown. (**B**) Thymocytes of WT, HET, and KO Aiolos mice were intracellularly stained with anti-Aiolos antibodies or kept unstained and analyzed by flow cytometry. Aiolos expression in iNKT cells is shown (*n* = 3). (**C**) iNKT cells were analyzed by staining thymocytes and splenocytes from WT, HET, and KO mice with CD1dt and anti-TCRβ antibodies. Total and relative cell numbers are depicted (*n* = 6). (**D**) Different stages of thymic iNKT cell development were analyzed using anti-CD24, anti-CD44, and anti-NK1.1 antibodies as in Fig. 4. Relative and total cell numbers are depicted (*n* = 4 to 10). (**E** and **F**) CD5 and TCR surface expression levels were measured in thymic iNKT cells from Aiolos WT and KO mice (*n* = 6).

Aiolos expression was reduced in the Kidins220 T-KO iNKT cells. We next asked whether a reduced Aiolos expression itself would result in a similar phenotype as the Kidins220 T-KO. This would be a hint that Aiolos could be a crucial downstream effector of Kidins220. Hence, we studied a germline Aiolos KO mouse strain where exon 8 was deleted, similar to a previously published line (Fig. 7A) (*66*). As expected, the protein Aiolos was not detectable in the KO iNKT cells but was present in the WT cells (Fig. 7B). The iNKT cells of the heterozygous (HET) mice showed intermediate expression levels. iNKT cell numbers were reduced in the thymus and spleen of Aiolos KO mice (Fig. 7C), resembling the Kidins220 T-KO. The HET cell numbers were in between. The NK1.1^+^ iNKT cell numbers were reduced in the spleen and not the NK1.1^−^ ones (fig. S8A). This could indicate that similar to the Kidins220 T-KO, the iNKT1 cells were more affected than other iNKT lineages in the Aiolos KO.

Focusing on the development of the iNKT cells in the thymus, the stage 0 iNKT cells were equal in number (and percentwise increased) in the Kidins220 T-KO compared to the Ctrl (Fig. 4, A and B). The same was true in the Aiolos KO (Fig. 7D). At stage 3, the iNKT cell numbers were strongly reduced in the Kidins220 T-KO (Fig. 4A) and in the Aiolos KO (Fig. 7D). As in the Kidins220 T-KO iNKT cells, there was an enhanced CD5 expression level in the iNKT cells in the Aiolos KO compared to the WT (Fig. 7E), suggesting that in the absence of Aiolos, TCR signaling was slightly enhanced. The stronger signaling might have led to increased apoptosis in the Aiolos KO iNKT cells (fig. S8B), although the increase was not statistically significant and not as pronounced as in the Kidins220 T-KO (Fig. 5, A and B). This substantiates that Aiolos is not the only downstream effector of Kidins220.

In contrast to the Kidins220 T-KO, the TCR expression levels in the Aiolos KO were not enhanced compared to the WT (Fig. 7F and fig. S8C), suggesting that enhanced TCR levels were not the cause for the iNKT phenotype in the Kidins220 T-KO mice.

As in the Kidins220 T-KO mice, the development of conventional T cells was only affected slightly in the Aiolos KO (fig. S9). This suggests that Aiolos function is more important for iNKT cells than for the conventional T cells. In conclusion, Aiolos might be a so far unknown downstream effector of Kidins220 in iNKT cell development in the thymus.

## DISCUSSION

Here, we show that the T-KO of Kidins220 leads to a severe reduction of iNKT and MAIT cell numbers, whereas the number of conventional T cells is only slightly reduced. Kidins220 reduces TCR signaling during iNKT cell development, allowing iNKT1 cells to develop and promotes TCR signaling in peripheral iNKT cells. Thus, we show here that, as a TCR-associated membrane protein, Kidins220 regulates TCR signaling in vivo. Last, we found that the transcription factor Aiolos is a downstream effector of Kidins220 in iNKT1 development.

During conventional T cell development, the numbers of T-KO DP cells in comparison to Ctrl decrease the further the cells progress. In the DP1 stage, which comprises the preselection thymocytes, the TCR is not signaling active (*49*). Cell numbers were not reduced at this stage in the T-KO, being in line with the idea that Kidins220 regulates mostly TCR signaling. In the DP2 stage, the cells were just positively selected by TCR signals (*49*) and DP3 cells are post-positive selection. At these two stages, reduced cell numbers were present in the T-KO, suggesting that positive selection was impaired in the absence of Kidins220. Consequently, also CD4^+^ and CD8^+^ SP cells, numbers were reduced in the T-KO.

Similarly, during the development of iNKT cells, TCR signals play a role; the TCR signal strength progressively decreases from stage 0 to stage 3 (*26*, *67*). Similar to others (*11*, *26*, *56*, *67*), we have used CD5 and Nur77 expression as readouts for TCR signal strength to evaluate the role of Kidins220 in iNKT cell development. In stage 0, the TCR signal strength appeared similar in Ctrl and T-KO cells. Likewise, the transcriptomes of Ctrl and T-KO cells were nearly identical in stage 0, suggesting that Kidins220 does not play a strong role at this stage. We did not find reduced cell numbers in stage 0 in T-KO mice (and also not in the different subpopulations of stage 0 as identified by scRNA-seq).

After stage 0, the TCR signal strength steers development into the different iNKT subsets. iNKT1 cells, which require a weak TCR signal to develop (*35*), were affected most by the T-KO and were reduced 10-fold. The remaining iNKT1 cells exhibited increased TCR signaling (CD5 and Nur77) (stage 3 cells are mostly iNKT1 cells) and enhanced apoptosis. We also found increased proliferation in stage 3 iNKT cells, but this could not compensate for the enhanced apoptosis. The generation of iNKT17 cells is promoted by TCR signals of medium strength, and their cell numbers were similar in T-KO and Ctrl. The generation of iNKT2 cells benefits from strong TCR signals and was slightly diminished (twofold). In support of our finding, it was already shown that stronger TCR signaling in iNKT cells leads to a slight reduction of iNKT2 cell numbers (*67*).

Kidins220 shows progressively higher expression levels from stage 1 to stage 3 (www.immgen.org/), supporting the assumption that in later stages of development, Kidins220 is needed the most to dampen TCR signals. This would make iNKT1 cells, which are mostly stage 3 cells, the most Kidins220-dependent iNKT subtype—in line with our observations. Conversely, in the hypomorphic ZAP-70 mice, TCR signaling was reduced in stages 2 and 3, and iNKT1 cells were percentwise increased (*35*–*37*, *68*). Together, our data are a further support for the notion that iNKT cells receive persisting TCR engagement also at later stages (*30*, *69*). The phenotype of T-KO mice is very similar to the Slam family receptor (SFR)–deficient mice (*67*), suggesting that these molecules might play similar roles in iNKT cells. For example, stage 3 iNKT cell numbers were also diminished the most in SFR KO mice and TCR signaling was also elevated in stage 2 and 3 iNKT cells.

Analysis of the transcriptional signatures at single-cell resolution of stage 3 iNKT cells has revealed a large heterogeneity, indicating that within stage 3, subpopulations exist (*32*, *33*). Cells develop from iNKT1a to iNKT1b and iNKT1c. Staining for NK1.1 and Sca-1 revealed that T-KO cell numbers were increased at iNKT1a but reduced at the iNKT1b and iNKT1c states. This was confirmed by our scRNA-seq data, suggesting that Kidins220 has a strong function at iNKT1a, allowing the cells to progress to iNKT1b and preventing apoptosis at iNKT1b and iNKT1c. We found a number of differentially expressed genes at our iNKT1a-like cells (cluster 0) comparing Ctrl and T-KO. Thus, these genes are downstream of Kidins220. Deleting one of those, namely, Aiolos, led to very similar phenotype as the Kidins220 KO: iNKT cell numbers were reduced, especially at stage 3, and CD5 levels were increased in the thymus. Thus, Aiolos is a downstream effector of Kidins220.

CD1d, the ligand for the iNKT TCR, is expressed at slightly lower levels in the T-KO mice. Using bone marrow chimeric mice in which T-KO iNKT precursors have access to sufficient amounts of CD1d, we showed that iNKT cell numbers were also diminished, substantiating our conclusion that it is an iNKT cell intrinsic mechanism that led to reduced iNKT cell numbers, namely, an altered TCR signal strength caused by the absence of Kidins220. The enhanced TCR signaling that we found in the T-KO cannot be explained by lower CD1d levels.

In peripheral iNKT cells, Kidins220 has a positive regulatory function. TCR signaling as measured by Nur77 expression was reduced after stimulation with αGalCer in splenic T-KO iNKT cells. This resulted in lower iNKT cell activation measured as reduced expression of CD69 and IL-4. Likewise, the ex vivo activation of peripheral conventional T cells was reduced in the absence of Kidins220. This is in line with data derived from a murine T cell line, in which TCR signaling was reduced when Kidins220 was knocked down (*38*), and from cardiovascular and neurological systems, where Kidins220 positively couples several receptors to intracellular signaling (*44*). A changing role of Kidins220 was also shown in the nervous system when triggering TrkB. In embryonic astrocytes, Kidins220 promotes kinase-based signaling and after birth Ca^2+^-dependent signaling (*70*).

In conclusion, our data show that Kidins220 regulates TCR signaling and we have no evidence that it would regulate signaling by other receptors. With which mechanisms does Kidins220 control TCR signaling in iNKT cells? In theory this might be done at three steps; first, by controlling TCR expression levels; second, by coupling to different signaling proteins; and last, by changing the transcriptome of the cells so that TCR signaling is reduced.

First, Kidins220 is involved in regulating receptor levels on the cell surface. When Kidins220 was down-regulated in neurons or in a T cell line, glutamate receptor 1 or TCR levels were increased, respectively (*38*, *71*). Likewise, TCR levels were slightly increased in T-KO compared to Ctrl in stage 3 thymic and in splenic iNKT cells. Because TCR signals were enhanced in stage 3 iNKT cells but reduced in splenic iNKT cells, TCR levels might not play the main role. In the Aiolos KO cells, TCR levels were not changed, but the same phenotype as in the Kidins220 KO was seen, including enhanced CD5 expression in iNKT cells. Thus, Kidins220 regulates TCR signaling beyond TCR expression levels.

Second, it was shown that Kidins220 binds to the TCR and couples the TCR to downstream signaling (*38*). Binding to and coupling receptors to signaling pathways is a general function of Kidins220, as it was also demonstrated in B cells and neurons (*39*, *44*, *46*, *70*, *72*).

Last, in the iNKT cells from Aiolos KO mice, TCR signaling, as measured by CD5 levels, was enhanced. Because Aiolos is a transcription factor (*65*), this suggests that genes are differently expressed by Aiolos to reduce TCR signaling. This might also hold true for the Kidins220 KO because less Aiolos is expressed in the Kidins220 KO. In our transcriptome data of stage 3 cells, we found a large number of signaling proteins being differentially regulated in the Kidins220 KO versus the Ctrl (table S2), including *Dusp1*, *Gpr171*, *Jun*, *JunB*, and *Fos* involved in TCR signaling or *Cited2*, *Tmem173*, and *Slfn2* implicated in the nuclear factor κB pathway. However, the increase in signaling and in apoptosis was not as pronounced in the Aiolos KO compared to the Kidins220 T-KO, suggesting that Kidins220 diminishes signaling by additional means than Aiolos expression.

MAIT cells were also strongly reduced in numbers in the Kidins220 KO, supporting the finding that in mice, the development of iNKT and MAIT follows identical paths (*34*). In the absence of functional Helios, another Ikaros family member, MAIT cell, was reduced in humans (*73*). Thus, the Kidins220-Helios/Aiolos axis might operate in iNKT and MAIT cells in mice and humans.

Our data also shed light on the generation of CD4^−^ and CD4^+^ iNKT1 cells. iNKT1a cells are mostly CD4^+^. In the steps to iNKT1c, CD4 is lost until the number of CD4^+^ and CD4^−^ cells is similar. In the iNKT1a state, the ratios of CD4^+^ to CD4^−^ cells are equal in T-KO and Ctrl. Only in iNKT1b and iNKT1c, T-KO cell numbers are decreasing compared to Ctrl, with CD4^−^ being reduced more than CD4^+^ cells, showing that Kidins220 promotes the generation of CD4^-^ iNKT cells. This suggests that weak TCR signals promote the generation of CD4^−^ iNKT1 cells.

One limitation of this study is that we did only obtain scRNA-seq data of Ctrl and T-KO cells from stage 1 and 2 iNKT cells. Thus, we cannot follow the development of T-KO cells at the transcriptome level.

In conclusion, we show that the scaffold protein Kidins220, which binds to the TCR, regulates the TCR signaling during development of iNKT cells. Thus, in the absence of Kidins220, developing thymic iNKT cells receive too strong signals and die by apoptosis, thus limiting the number of iNKT cells. Part of this Kidins220 function might be mediated by Aiolos. Because iNKT1 cells are more affected than iNKT2 or iNKT17 cells, our data are in line with earlier findings that iNKT1 cells require weak TCR signals to develop (*35*). Because Kidins220 binds to many receptors in different cell types (*39*, *44*, *46*, *70*, *72*), our findings might serve as a blueprint to re-examine signal transduction by other receptors.

## MATERIALS AND METHODS

### Experimental design

The aim of this study was to uncover the in vivo role of Kidins220 in the development and activation of T cells in mice. By binding to the TCR, we hypothesized that Kidins220 would alter the development of conventional T cells and iNKT cells. To this end, we generated a T cell–specific Kidins220 KO mouse strain and analyzed the T cells after extraction from the mice by various techniques, including flow cytometry and scRNA-seq. Furthermore, we stimulate iNKT cells in vivo by injecting their TCR ligand into the mice. Last, we also analyzed Aiolos KO mice because we found that Aiolos was downstram of Kidins220.

### Mice

Kidins220^+/flox^ mice (*45*) provided by G. Schiavo (University College London, London, England, UK) were crossed to pLckCre mice (Kidins220lckCre mice). Mice were between 6 and 46 weeks old but for most experiments between 9 and 15 weeks. The C57BL/6Rag2^−/−^ (Rag2 KO), C57BL/6-Ly5.1 (CD45.1), and Kidins220lckCre mice were bred under specific pathogen–free conditions. All mice were maintained in C57BL/6 background. Mice were sex- and age-matched with litter controls whenever possible. Mice were backcrossed minimum 10 generations to C57BL/6. All animal protocols (G19/151) were performed in accordance with the German animal protection law with authorization from the Veterinär- und Lebensmittelüberwachungsbehörde, Freiburg, Germany.

The Aiolos *Ikzf3* null allele was generated by CRISPR-Cas9–mediated deletion of the sequences between the chr11:98,466,968 and chr11:98,467,878 genomic positions (mm10) via electroporation of appropriate guide RNAs and the Cas9 protein into fertilized eggs (C57BL/6N). This deletion removes the 3′ part of intron 7 and the coding sequence of exon 8 and was targeted as published before (*66*). Mice were bred in a Specific pathogen–free facility and studied at 6 to 9 weeks of age according to Institute of Genetics, Molecular and Cellular Biology Ethical Committee (Com’Eth) guidelines.

### Flow cytometry

To gain single-cell suspensions from thymus or spleen, the organs were mechanically disrupted. Lungs were cut with scissors and treated with collagenase P (1 mg/ml) and deoxyribonuclease 1 (0.1 mg/ml) at 37°C for 1 hour. Afterward, the digested lungs were forced through a 70-μm strainer. Single cells are the obtained by gradient centrifugation using Percoll. Livers were cut with scissors and forced through a 70-μm strainer. Hepatocytes were gained by centrifugation with Percoll supplemented with heparin (100 U/ml). Erythrocytes were lysed using Ammonium-Chloride-Potassium lysis buffer (150 mM NH_4_Cl and 10 mM KHCO_3_) in all single-cell suspensions. Afterward, the cells were stained. Flow cytometry was performed as shown in the company’s instructions using a Gallios (Beckam Coulter) or LSRFortessa (BD Biosciences). Data evaluation was performed using FlowJo X.

### Antibodies and tetramers

The following antibodies were used to stain cells for flow cytometry: phycoerythrin (PE)–labeled anti-CD1d (1B1), Peridinin-Chlorophyll-Protein (PerCP)–Cy5.5–labeled anti-CCR7 (4B12), PE-Cy7–labeled anti-CD44 (IM7), PE-labeled anti-CD45.2 (104), fluorescein isothiocyanate (FITC) Annexin V Apoptosis Detection Kit I, FITC BrdU Flow Kit, AF647-labeled anti-Nur77 (12.14), and anti-Aiolos (S48-791) were purchased from BD Biosciences. Allophycocyanin (APC)–labeled anti-CD122 (TM-β1), Pacific Blue (PB)-labeled anti-CD19 (6D5), FITC-labeled anti-CD45.1 (A20), FITC-labeled anti-IFNγ (XMG1.2), PE-Cy7–labeled anti–IL-4 (11B11), PE-labeled anti-MR1 (26.5), FITC-labeled anti-NK1.1 (PK136), PE-Cy7–labeled anti-Sca-1 (D7), PE-labeled anti-Tbet (4B10), FITC-labeled anti-TCRβ (H57-597), and PB-labeled anti-TCRβ (H57-597) were purchased from BioLegend. PerCP-Cy5.5–labeled anti-CD24 (M1/69), FITC-labeled anti-CD4 (GK1.5), PE-labeled anti-CD4 (RM4-5), PE-Cy7–labeled anti-CD4 (RM4-5), APC-labeled anti-CD44 (IM7), APC-labeled anti-CD5 (53-7.3), PE-Cy7–labeled anti-CD69 (H1.2F3), eFluor660-labeled anti-CD8α (53-6.7), anti-CD3 (145-2C11), and APC-labeled anti-TCRβ (H57-597) were purchased from eBioscience. PE-Cy7–labeled anti-NK1.1 (PK136) was purchased from Invitrogen. CellEvent Caspase-3/7 green flow cytometry assay kit was purchased from Thermo Fisher Scientific. The anti-BCL_XL_ antibody (54H6) was from Cell Signaling Technology. CD1d tetramers and MR1 tetramers were provided by the NIH Tetramer Core Facility.

### Apoptosis assays

To analyze relative amounts of apoptotic cells, the cells were cultured in RPMI medium supplemented with 10% fetal bovine serum (FBS) for 18 hours before analysis. After staining with surface antibodies, the cells were treated with the Caspase 3/7 reagent (1:200) for 45 min on room temperature (CellEvent Caspase-3/7 green flow cytometry assay kit). This reagent comprises a nucleic acid–binding dye coupled to a peptide (DEVD), which is cleaved by active Caspase 3/7. After cleavage, the dye enters the nucleus and stains nucleic acids, enabling the detection of apoptotic cells. For analyzing apoptosis with annexin V (1:200), it was added for 15 min on room temperature (FITC Annexin V Apoptosis Detection Kit I). For both approaches, after incubation, the cells were flow cytometrically analyzed without washing. Dead cells were excluded on the basis of forward scatter (FSC) and side scatter (SSC) intensities.

### Mixed bone marrow chimera

Bone marrow cells were isolated from CD45.1^+^ mice and mixed in a 1:1 ratio with either isolated bone marrow cells from CD45.2^+^ Ctrl or CD25.2^+^ T-KO mice. Ten million cells of mixed bone marrow cells were intravenously injected into lethally irradiated (9.5 gray) Rag2 KO mice. Chimeric mice were euthanized, and relative iNKT cell numbers were analyzed 7 to 8 weeks after injection.

### In vivo BrdU assay

BrdU (1.5 mg) diluted in 200 μl of phosphate-buffered saline (PBS) were intraperitoneally injected into Ctrl and T-KO mice. Mice were euthanized 16 or 24 hours after injection. Thymocyte proliferation of BrdU-treated mice was assessed following the FITC BrdU Flow Kit (BD) protocol.

### In vivo αGalCer challenge

To test antigen response of iNKT cells in vivo, 5 μg of αGalCer (purchased from Biozol) dissolved in 200 μl of PBS supplemented with 5.6% sucrose, 0.75% l-histidine, and 0.5% Tween 20 or only buffer was intraperitoneally injected into Ctrl and T-KO mice. Two hours after injection, mice were euthanized and splenocytes were isolated to analyze the antigen response of splenic iNKT cells. For this, intracellular cytokines were analyzed as described in the “Intracellular staining of cytokines and transcription factors” section.

### Ex vivo T cell stimulation

Splenocytes of Ctrl and T-KO mice were stimulated for 4 hours at 37°C and 5% CO_2_ in complete RPMI 1640 medium with 10% FBS using anti-CD3 (5 μg/ml; 145-2C11) and anti-TCRβ (5 μg/ml; H57) antibodies. As control, cells were also left unstimulated for the 4 hours. Subsequently, cells were stained with anti-CD4, anti-CD8, and anti-CD25 antibodies and analyzed by flow cytometry.

### Intracellular staining of cytokines and transcription factors

To detect intracellular cytokines and activation markers via flow cytometry, the Cytofix/Cytoperm Kit (BD) was used. Fluorescently labeled CD1dt and surface antibodies were stained as usual. Following to this, cells were fixed, permeabilized, and stained with anti–IFN-γ, anti–IL-4, anti-BCL_XL_ and anti-Nur77 antibodies in Perm/Wash buffer following the manufacturer’s protocol of the Cytofix/Cytoperm Kit. To stain transcription factors, fluorescently labeled CD1dt and antibodies were used to stain surface molecules. Furthermore, fixation and permeabilization were done according to the manufacturer’s protocol of the Foxp3/Transcription Factor Staining Buffer Set (eBioscience). Following, cells were incubated with transcription factor antibodies (FoxP3, Tbet, and Aiolos) and analyzed by flow cytometry.

### SDS-PAGE and Western blotting

Total thymocytes were lysed as before and separated by reducing SDS–polyacrylamide gel electrophoresis (SDS-PAGE) (*74*). Protein transfer was performed using wet tank transfer onto polyvinylidene difluoride membranes. After blocking, membranes were incubated with anti-Kidins220 (rabbit, 21856, Proteintech) and anti-glyceraldehyde-3-phosphate dehydrogenase (rabbit, G9545, Sigma-Aldrich) antibodies and after washing with a horse radish peroxidase-coupled anti-rabbit secondary antibody. Signals were detected using the ImageQuant LAS 4000mini system (GE Healthcare).

### Polymerase chain reaction

DNA of splenocytes was used for a polymerase chain reaction using the primers GAGCACAGACTTCTCTTATGG (long, forward), GCGTTTCTAGCATACACATG (flox, reverse), and CAGATGGCTGTGAACCACCGTTTAAAC (slice, reverse) with an annealing temperature of 55°C and 35 cycles.

### Single-cell RNA sequencing

To perform the scRNA-seq experiment, cells of four thymi of Ctrl mice were pooled and the same was done with four thymi of T-KO mice. Cells were stained with CD1d tetramers, anti-TCRβ, anti-NK1.1, anti-CD44, and anti-CD24 antibodies on ice. Cells were sorted by flow cytometry for TCRβ^+^, CD1dt^+^, CD24^+^, and NK1.1^−^ cells to obtain stage 0 iNKT cells as well as for TCRβ^+^, CD1dt^+^, NK1.1^+^, CD24^−^, and CD44^+^ cells to obtain stage 3 iNKT cells. This was done twice in parallel to have two replicas each. After sorting, each cell population was barcoded by hash-tagged antibodies (TotalSeqC format, anti-mouse Hashtag 1 to 8; clone M1/42, 30-F11, BioLegend). The antibody concentrations used were 1 μg per million cells. After staining, cells were washed three times in PBS containing 2% bovine serum albumin and 0.01% Tween 20, followed by centrifugation (300*g* for 5 min at 4°C) and supernatant exchange. Then, all eight populations were pooled (2× stage 0 Ctrl, 2× stage 0 T-KO, 2× stage 3 Ctrl, and 2× stage 3 T-KO). Sorted cells were processed through the 10X Genomics single-cell V(D)J workflow according to the manufacturer’s instructions. Libraries were pooled to desired quantities to obtain appropriate sequencing depths as recommended by 10X Genomics and sequenced on a NovaSeq 6000 flow cell.

### Quantification of gene expression and protein abundance

Quantification of gene expression and hashtag protein abundance counts was performed using cellranger-6.0.0 using the count command, which performs alignment, filtering, barcode counting, and unique molecular identifier counting as well as process feature barcoding data. Alignments were performed using prebuilt Cell Ranger and Cell Ranger ARC mouse mm10 references.

### scRNA-seq data analysis

scRNA-seq data was analyzed using Seurat (*61*). Low-quality cells having mitochondrial percentage greater than 5 and number of genes per cell less than 1000 were removed from the analysis. Ribosomal genes (small and large subunits) and predicted genes with Gm-identifier were excluded from the analysis. The normalization method was set to “LogNormalize”. Top 2000 variable features were used for clustering analysis. Dimensionality reduction was performed using the RunUMAP function, where dims was set to 1:30. Default resolution was used for clustering. To characterize the clusters, differential gene expression analysis was performed using the FindMarkers function in Seurat. We removed certain clusters from the analysis. In the first clustering analysis, we detected B cells, myeloid cells, DP, and DN thymocytes. The latter might have been present due to the poor CD1dt stain of our cells in that particular sort (fig. S6A). Those cells were removed. We also had two clusters composed of 15 and 50 cells that were iNKT17 cells and iNKT cells of unknown identity, respectively. Because of the low cell numbers, these two clusters were further removed. Trajectory inference (pseudotime analysis) was performed using Monocle 3 with default settings (*75*). Cluster 0 was used as the root cell cluster for pseudotime inference.

### Statistical analysis

For cell numbers and MFIs, the statistical analysis was done by two-sided Student’s *t* test and for relative cell number values by Mann-Whitney *U* test. Concerning the scRNA-seq data, a paired analysis of total cell numbers was done by Wilcoxon matched-pairs signed rank test. *P < 0.05, **P < 0.01, ***P < 0.001, and ****P < 0.0001. Error bars indicate SEM.
