# Supplementary material for: Kidins220 and Aiolos promote thymic iNKT cell development by reducing TCR signals
Source: Sci Adv. 2024 Mar 15;10(11):eadj2802. doi: 10.1126/sciadv.adj2802 (PMC10942104; doi:10.1126/sciadv.adj2802)
Supplement: Supplementary file 1 — Figs. S1 to S9 [file sciadv.adj2802_sm.pdf]

Science Advances
AAAS

Supplementary Materials for

Kidins220 and Aiolos promote thymic iNKT cell development by reducing TCR signals

Laurenz A. Herr *et al.*

Corresponding author: Wolfgang W. Schamel, wolfgang.schamel@biologie.uni-freiburg.de

Sci. Adv. **10**, eadj2802 (2024)
DOI: 10.1126/sciadv.adj2802

The PDF file includes:

Figs. S1 to S9

Other Supplementary Material for this manuscript includes the following:

Tables S1 and S2

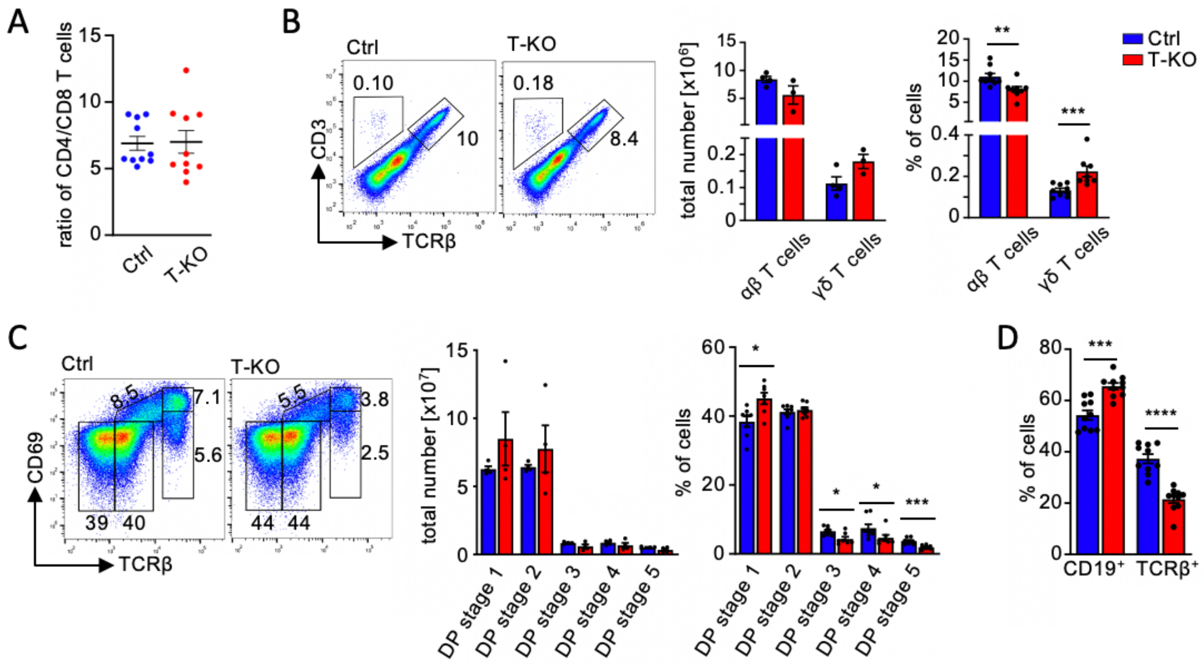

Fig. S1. Thymic T cell development in Kidins220 T-KO mice.
(A) The ratio of the numbers of CD4$^+$ to CD8$^+$ SP cells in the thymus is displayed. (B) Thymocytes were stained with anti-TCRβ and anti-CD3 antibodies to analyze total cell numbers and percentages of αβ T cells and γδ T cells (n = 8 for percent of cells; n = 3 for total cell numbers). (C) Analysis of thymocyte development (gated on total thymocytes) was done using anti-TCRβ and anti-CD69 antibodies (stage 1: TCRβ^{low}CD69^{low}; stage 2: TCRβ^{int}CD69^{low}; stage 3: TCRβ^{int}CD69^{int}; stage 4: TCRβ^{high}CD69^{high}; stage 5: TCRβ^{high}CD69^{int}). The graphs show relative values as well as total cell numbers (n = 10). (D) B cells and T cells in the spleen of Ctrl and T-KO mice were analyzed by anti-CD19 and anti-TCRβ staining. Relative values are shown (n = 7-10). These are the same data as shown in figure 1E.

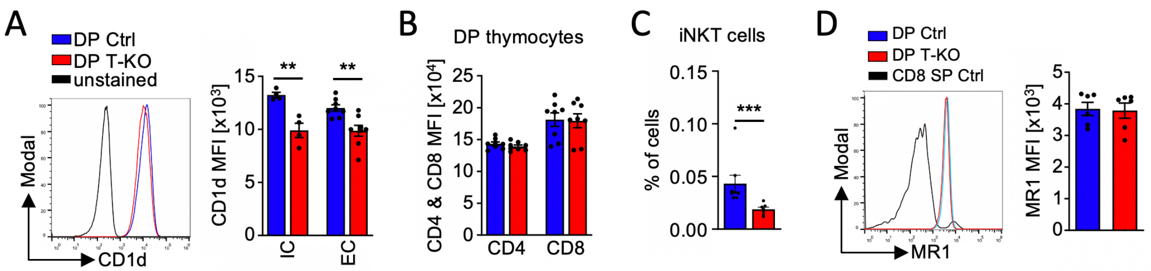

Fig. S2. CD1d, CD4, CD8 and MR1 levels on DP thymocytes.
(A) The CD1d expression level on DP thymocytes was analyzed by intracellular (IC) or surface (EC) stains using anti-CD1d antibodies and flow cytometry. Bar diagrams depict the MFI (n = 4 for IC, n = 8 for EC stains). (B) CD4 and CD8 expression levels on the surface of DP thymocytes were analyzed by stains using anti-CD4 and anti-CD8 antibodies and flow cytometry (n = 8). (C) Bone marrow cells from CD45.1$^+$ WT mice were mixed with bone marrow cells of either CD45.2$^+$ Ctrl or CD45.2$^+$ T-KO mice in a 1:1 ratio and injected into lethally irradiated Rag2 KO mice. The bar diagram shows relative numbers of CD45.2$^+$ Ctrl (blue) and T-KO (red) of thymic iNKT cells (n = 7 - 8). (D) Thymocytes were stained with anti-CD4, anti-CD8 and anti-MR1 antibodies and analyzed by flow cytometry. Mean fluorescence levels of MR1 on DP cells of Ctrl and T-KO cells are shown (n = 6). As control, MR1 levels on CD8 SP cells is shown.

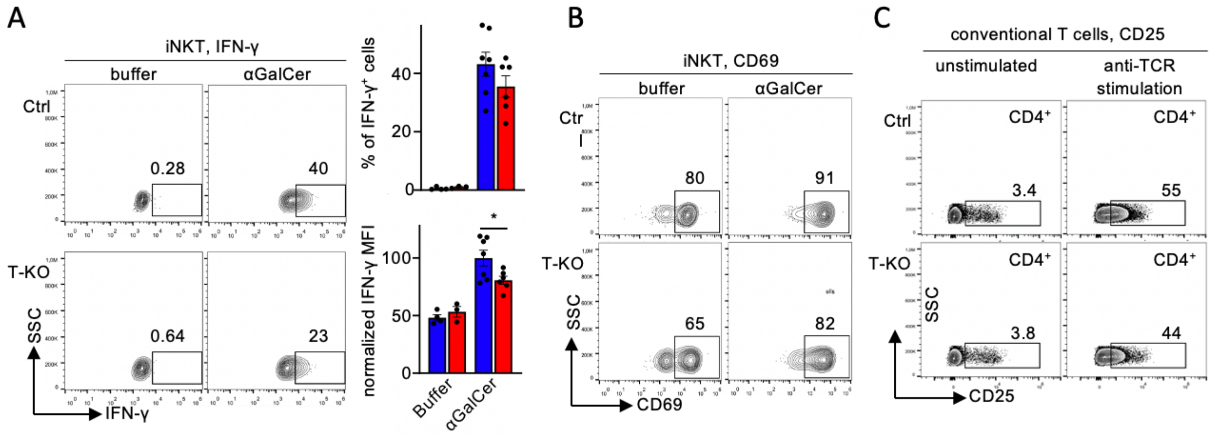

Fig. S3. Splenic T-KO iNKT cells are less responsive to αGalCer.
(A, B) Ctrl and T-KO mice were killed 2 h after intraperitoneal injection of αGalCer. Splenocytes were analyzed for (A) IFN-γ$^+$ iNKT cells, IFN-γ expression levels as well as for (B) CD69$^+$ iNKT cells and CD69 MFI by flow cytometry. Plots were pre-gated on CD1dt$^+$ TCRβ$^+$ iNKT cells (n = 6). (C) Splenic T cells from Ctrl and T-KO mice were stimulated for 4 hrs using anti-CD3 and anti-TCRβ antibodies and then stained with anti-CD4, anti-CD8 and anti-CD25 antibodies and analyzed by flow cytometry (n = 6). These are the original CD4$^+$ T cell data from the graph in figure 3E.

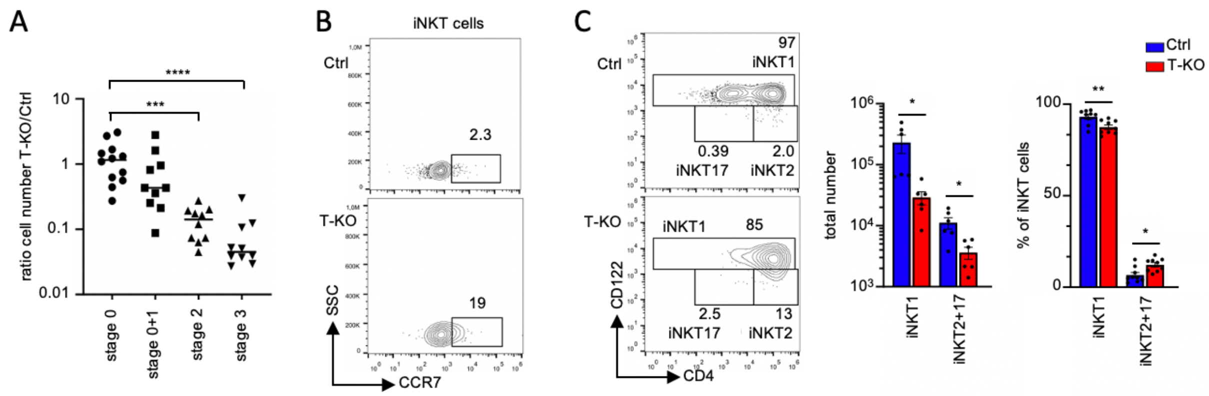

Fig. S4. iNKT1 cell (CD122$^+$) numbers are severely reduced in T-KO mouse thymi.
(A) The ratio of the cell numbers of T-KO and Ctrl mice was calculated for each stage separately. Data are those from figures 4A and B. (B) The expression of CCR7$^+$ identifying iNKT precursor cells is depicted (n = 6). The statistics are shown in figure 4C. (C) iNKT cell subsets were analyzed using anti-CD122 and anti-CD4 antibodies. iNKT1 cells are CD122$^+$ whereas iNKT2 cells and iNKT17 cells are CD122$^-$ CD4$^+$ and CD122$^-$ CD4$^-$, respectively. Samples were pre-gated on CD1dt$^+$ TCRβ$^+$ iNKT cells. Graphs show cell numbers as well as relative numbers (n = 6-9).

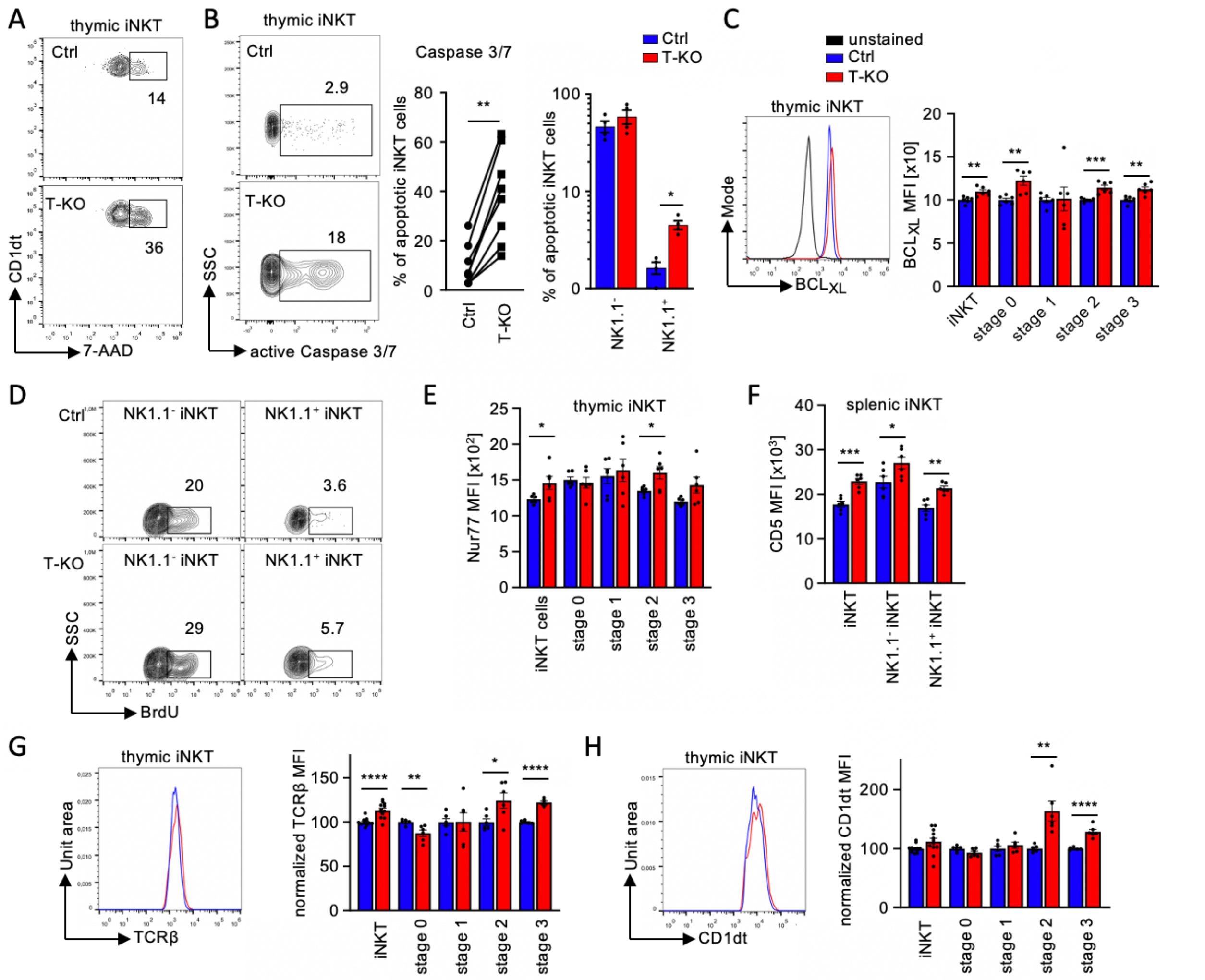

Fig. S5. Thymocytes of T-KO mice show a higher susceptibility to apoptosis, increased proliferation and enhanced TCR signaling compared to the ones of Ctrl mice.

(A) Dead thymocytes were detected using 7-AAD by flow cytometric analysis. iNKT cells were gated as CD1dt^{+} TCRβ^{+} cells (n = 6). These are the data of figure 5B. (B) To quantify apoptosis in iNKT cells, total thymocytes were cultivated for 18 h in RPMI medium supplemented with 10% FBS. Dead cells were excluded based on FSC and SSC values. Graph shows percentages of CD1dt^{+} TCRβ^{+} iNKT cells with active Caspase 3 and 7 (middle panel, n = 8). Apoptotic iNKT cells were subdivided into NK1.1^{-} and NK1.1^{+} cells (right panel, n = 4). (C) Total thymic iNKT cells and iNKT cells separated into the different stages as above were analyzed for BCL$_{XL}$ expression levels using anti-BCL$_{XL}$ antibodies in intracellular flow cytometric staining. Mean fluorescence intensities are depicted (n = 6). (D) These are the dot plots of the *in vivo* proliferation experiment of figure 5D. After BrdU injection into the mice thymocytes were stained with anti-BrdU antibodies. CD1dt^{+} TCRβ^{+} iNKT cells and iNKT cells which were subdivided into NK1.1^{+} and NK1.1^{-} iNKT cells were analyzed using flow cytometry (n = 6-7). (E) Nur77 MFI in iNKT cells as in figure 5E was determined by flow cytometric analysis using anti-Nur77 antibodies (n = 6). (F) CD5 MFI was assessed in splenic iNKT cells divided into NK1.1^{+} and NK1.1^{-} iNKT cells using anti-NK1.1 antibodies (n = 6). (G) TCRβ expression levels and (H) CD1dt binding were analyzed by pre-gating thymic cells on CD1dt^{+} TCRβ^{+} iNKT cells. iNKT stages were analyzed as above. MFIs are depicted (n = 6-12).

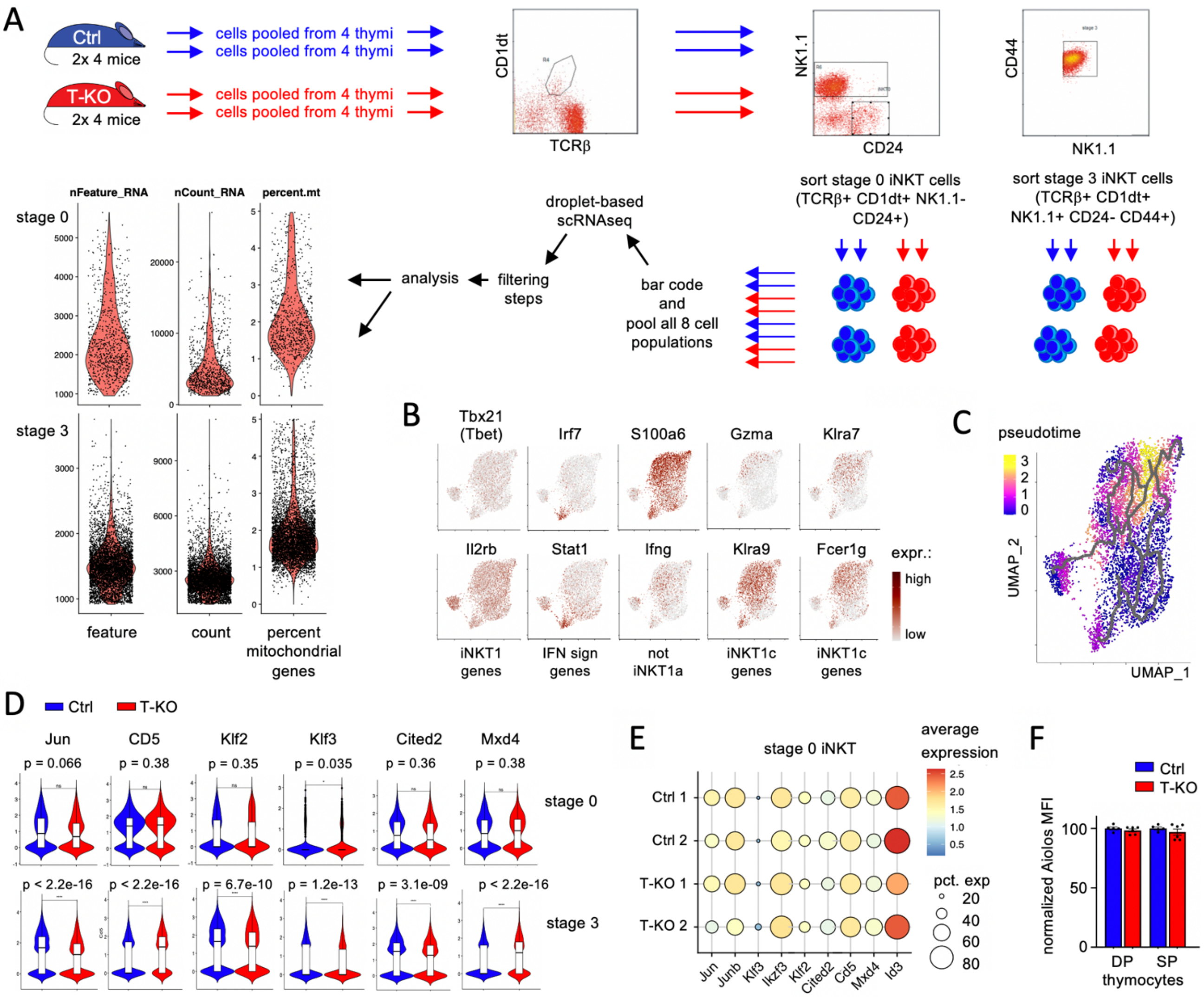

Fig. S6. scRNAseq of stage 0 and stage 3 iNKT cells sorted from Ctrl and T-KO mice. (A) Workflow of the scRNAseq procedure. (B) Expression of key marker genes used to classify the identity of the cells. (C) Trajectory analysis of the stage 3 scRNAseq data using Monocle 3. Color represents the pseudotime. Cells in cluster 0 were chosen as roots of the trajectory. (D) Violin plots included with box plots showing the normalized transcript counts of selected genes in stage 0 and stage 3 cells from Ctrl and T-KO. Significance was assessed using Wilcoxon test. (E) Dot plot showing the expression of 9 genes in stage 0 iNKT cells. Color represents the scaled expression of the gene in the respective condition and dot size represents the fraction of cells in the condition expressing the gene. Note that these genes were differentially expressed in stage 3, but are equally expressed in stage 0, comparing Ctrl to T-KO. (F) Ctrl and T-KO thymocytes were stained with an anti-TCRβ antibody and after fixation and permeabilization with an anti-Aiolos antibody and measured by flow cytometry. DP and SP cells were gated according to their TCRβ level and analyzed for Aiolos expression (n = 6).

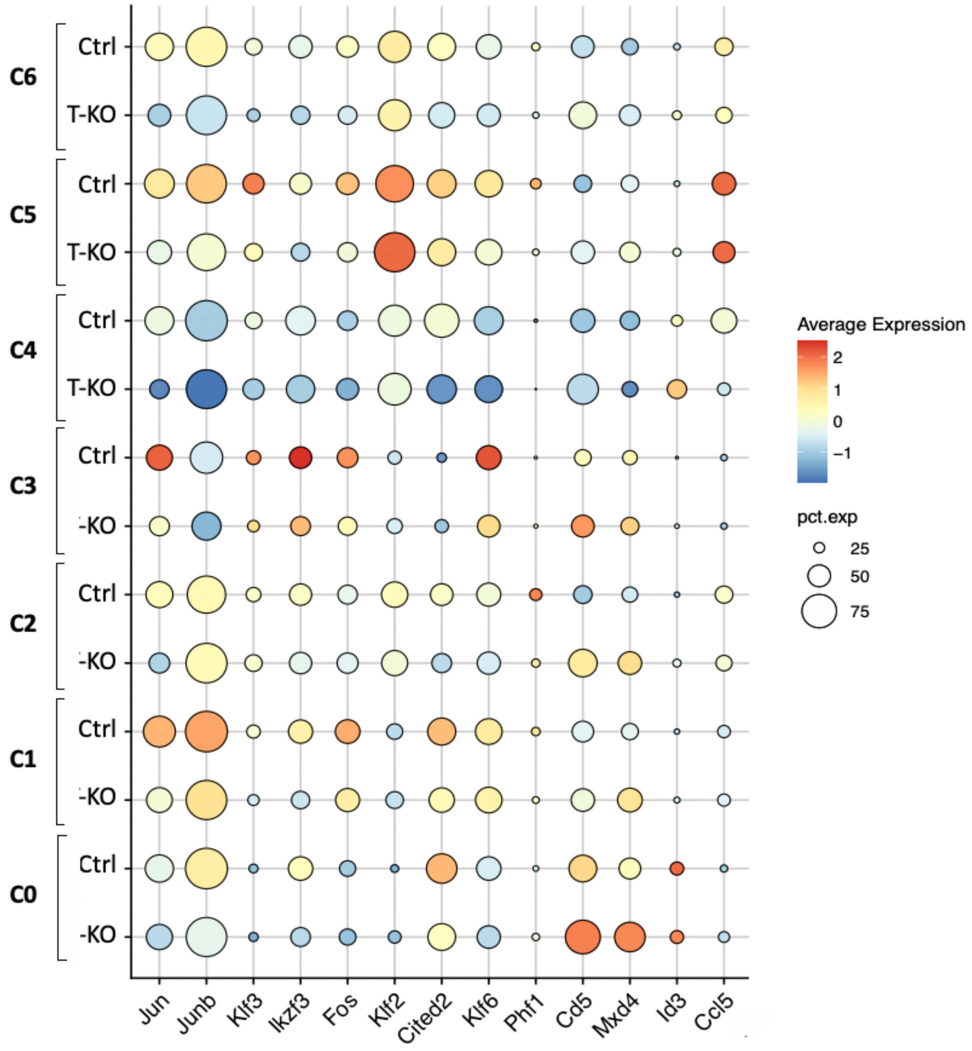

Fig. S7. Gene expression of stage 3 clusters for Crtl and T-KO.
Dot plot showing scaled expression of the 10 genes from figure 6H in all stage 3 clusters, from C0 to C6, separately for Ctrl and T-KO.

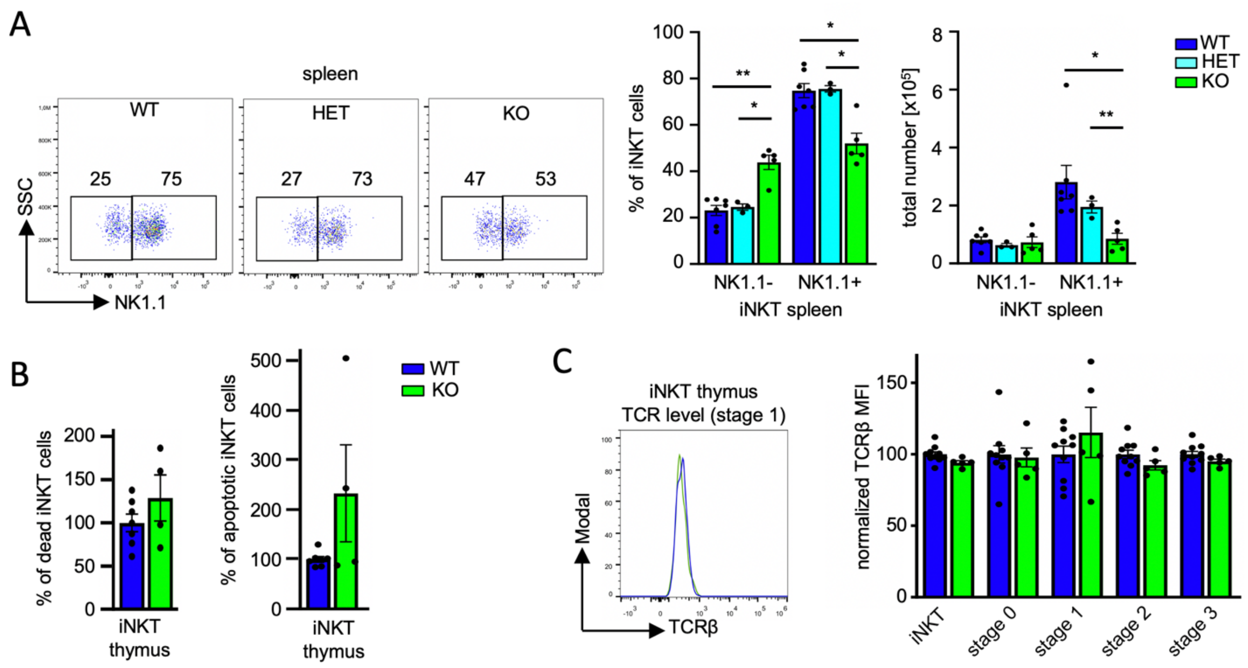

Fig. S8. Analysis of iNKT cells from thymus and spleen of Aiolos KO mice.
(A) iNKT cells were analyzed by staining splenocytes from WT, HET and KO Aiolos mice with CD1dt, anti-TCRβ and anti-NK1.1 antibodies. Histograms as well as relative and total cell numbers are depicted (WT, n = 7; HET, n = 3; KO, n = 5). (B) Dead and apoptotic iNKT cells were analysed as in figure 5A and 5B using thymocytes of WT and Aiolos KO mice. Graphs show the percentage of 7-AAD-positive cells (left; WT, n = 7; KO, n = 4) and the percentage of Annexin V^+ cells (right; WT, n = 7; KO, n = 4) both in CD1dt$^+$ TCRβ$^+$ iNKT cells. (C) TCRβ MFI in thymic iNKT cells of the different stages was assessed by flow cytometry using anti-TCRβ antibodies. The iNKT cells and stages were identified as above (WT, n = 10; KO, n = 5).

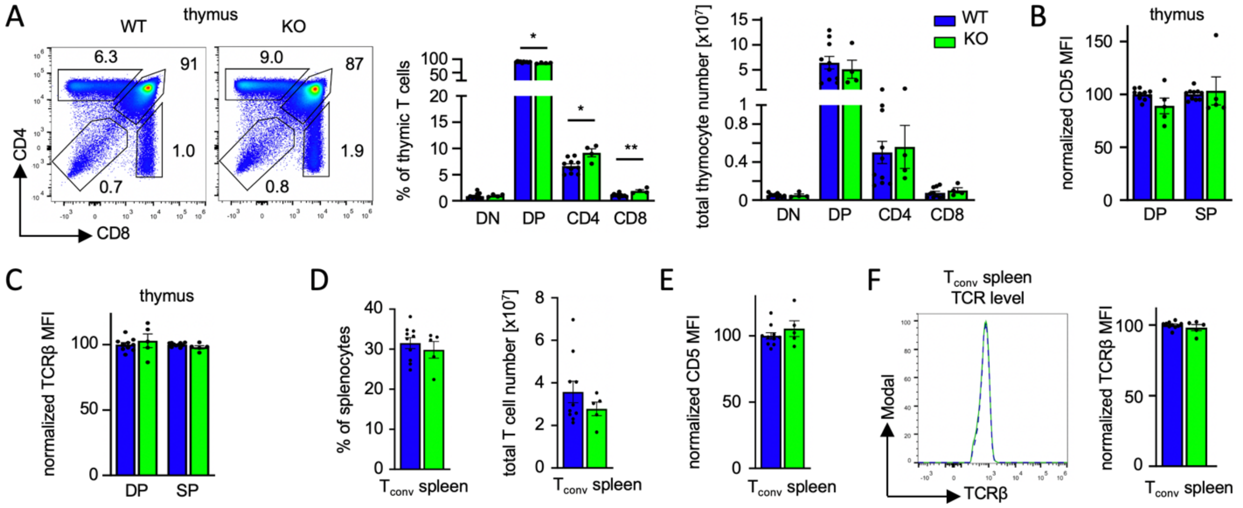

Fig. S9. Analysis of conventional thymic and splenic T cells of the Aiolos KO mice.

(A) Thymocyte development of WT and KO Aiolos mice was analyzed using anti-CD4 and anti-CD8 antibodies. Percentage of cells and total cell numbers are shown (WT; n = 10 and KO, n = 4). (B and C) CD5 MFI and TCRβ MFI in thymic DP and SP T cells of WT and Aiolos KO mice was assessed by flow cytometry using anti-CD5 and anti-TCRβ antibodies. DP and SP cells were gated according to their TCRβ level. The normalized MFIs for CD5 (B) and TCRβ (C) are displayed (WT, n = 10; KO, n = 5). (D) Splenocytes of WT and Aiolos KO mice were stained with anti-TCRβ antibodies. $TCR\beta^{+}$ cells were gated. Percentage of cells and total cell numbers are shown (WT, n = 10; KO, n = 5). (E and F) CD5 MFI and TCRβ MFI in splenic T cells of WT and Aiolos KO mice was assessed by flow cytometry using anti-CD5 and anti-TCRβ antibodies. $TCR\beta^{+}$ cells were gated. The normalized MFIs for CD5 (E) and TCRβ (F) are displayed (WT, n = 10; KO, n = 5).
